# Cerebrovascular reactivity has negligible contribution to haemodynamic lag after stroke: implications for fMRI studies

**DOI:** 10.1161/STROKEAHA.122.041880

**Published:** 2023-03-27

**Authors:** Andra Braban, Robert Leech, Kevin Murphy, Fatemeh Geranmayeh

**Affiliations:** 1 Clinical Language and Cognition group, Imperial College London, UK; 2 Centre for Neuroimaging Science, King's College London, UK; 3 Cardiff University Brain Research Imaging Centre, School of Physics and Astronomy, Cardiff University, UK

## Abstract

**Background:**

Functional MRI is ubiquitously used to study post-stroke recovery. However, the fMRI-derived haemodynamic responses are vulnerable to vascular insult which can result in reduced magnitude and temporal delays (lag) in the haemodynamic response function (HRF). The aetiology of HRF lag remains controversial, and a better understanding of it is required to ensure accurate interpretation of post-stroke fMRI studies. In this longitudinal study, we investigate the relationship between haemodynamic lag and cerebrovascular reactivity (CVR) following stroke.

**Methods:**

Voxelwise lag maps were calculated relative to a mean grey matter reference signal for 27 healthy controls and 59 patients with stroke across two timepoints (~2 weeks and ~4 months post-stroke), and two conditions: resting-state and breath-holding. The breath-holding condition was additionally used to calculate CVR in response to hypercapnia. HRF lag was computed for both conditions across tissue compartments: lesion, perilesional tissue, unaffected tissue of the lesioned hemisphere, and their homologue regions in the unaffected hemisphere. CVR and lag maps were correlated. Group, condition, and time effects were assessed using ANOVA analyses.

**Results:**

Compared with the average grey matter signal, a relative haemodynamic lead was observed in the primary sensorimotor cortices in resting-state and bilateral inferior parietal cortices in breath-holding condition. Whole-brain haemodynamic lag was significantly correlated across conditions irrespective of group, with regional differences across conditions suggestive of a neural network pattern. Patients showed relative lag in the lesioned hemisphere which significantly reduced over time. Breath-hold derived lag and CVR had no significant voxel-wise correlation in controls, or patients within the lesioned hemisphere or the homologous regions of the lesion and perilesional tissue in the right hemisphere (mean *r*<0.1).

**Conclusion:**

The contribution of altered CVR to HRF lag was negligible. We suggest that HRF lag is largely independent of CVR, and could partly reflect intrinsic neural network dynamics amongst other factors.

## Graphical abstract

**Figure:**
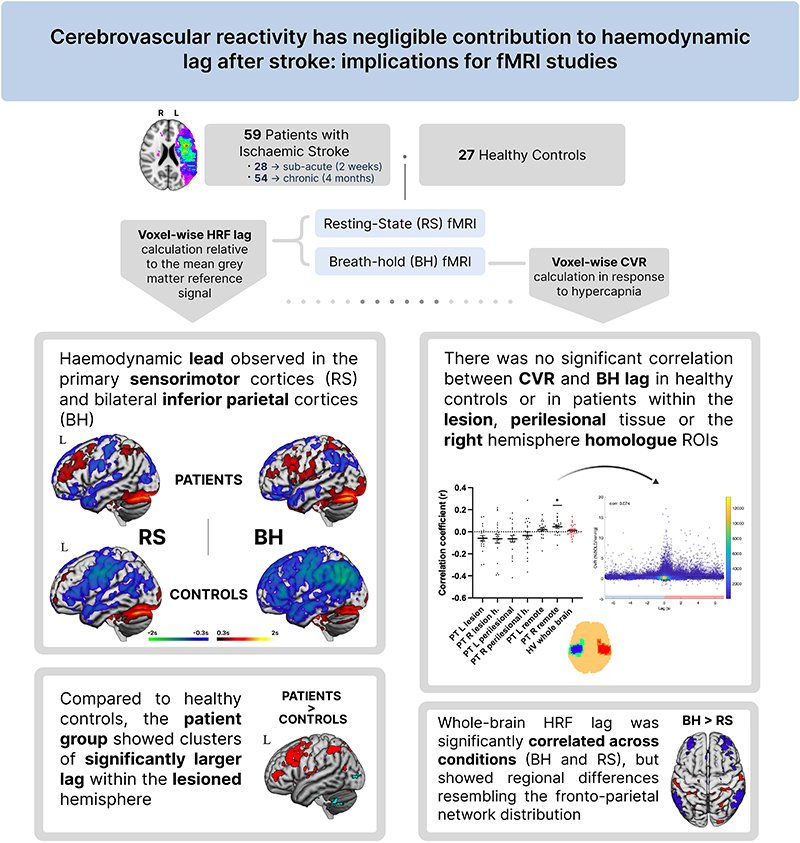

## Non-standard Abbreviations and Acronyms

aI/fO: anterior Insula/Frontal Operculum
BH: Breath-hold
BOLD: Blood Oxygen Level Dependent
CBF: Cerebral Blood Flow
CSF: Cerebrospinal Fluid
CVR: Cerebrovascular Reactivity
DOF: Degrees of Freedom
EPI: Echoplanar Imaging
FAST: FMRIB's Automated Segmentation Tool
FD: Framewise Displacement
FDR: False Discovery Rate
FEAT: FMRI Expert Analysis Tool
FLIRT: FMRIB's Linear Image Registration Tool
FMRI: Functional Magnetic Resonance Imaging
FSL: FMRIB's Software Library
GLM: General Linear Model
HRF: Haemodynamic Response Function
HV: Healthy Volunteers
IPL: Inferior Parietal Lobule
LME: Linear Mixed-Effect
MCA: Middle Cerebral Artery
PCC: Posterior Cingulate Cortex
PFC: Prefrontal Cortex
PT: Patients
ROI: Region of Interest
RS: Resting-state

## Introduction

Resting-state functional magnetic resonance imaging (fMRI) has become instrumental in enhancing our understanding of mechanisms of post-stroke functional recovery. The fMRI-derived BOLD (Blood Oxygen Level Dependent) signal is modelled through the haemodynamic response function (HRF) and is contingent on intact neurovascular coupling.^1^ Two key properties of the HRF are its temporal delay (lag) and magnitude. The former is calculated by cross-correlation with a reference signal (e.g., average grey matter signal) to derive a relative lead or lag, whilst the latter is used to measure cerebrovascular reactivity (CVR) in response to a vasoactive stimulus.

BOLD signal temporal delays, including a relative lead or lag, have been observed in health^2^ and after stroke.^3–7^ Specifically, resting-state temporal lags following stroke i) have been identified in areas of abnormal perfusion^3–5,8^, and positively correlate with stroke lesion size,^7^ ii) have been shown to extend beyond the lesion boundary in brain regions displaying altered haemodynamic flow,^3,6,7^ iii) may confound observed changes in functional connectivity,^6^ and iv) may explain variability in behavioural outcomes after stroke.^7^ Given the ubiquitous use of fMRI in post-stroke recovery literature and the vulnerability of BOLD signal to vascular insult, a better understanding of the mechanisms underpinning haemodynamic lag is needed for accurate interpretation of such studies in stroke.

Several factors have been proposed to contribute to haemodynamic lag observed in stroke which are not necessarily mutually exclusive. First, lag may be seen in indirectly affected regions that share a vascular supply with the lesioned area.^3,5,6^ Whilst some studies in patients with hyperacute^8^ or subacute^6^ stroke have demonstrated a correlation between perfusion metrics and haemodynamic lag, studies in large healthy cohorts, where observed lag is typically of lower magnitude, have not confirmed this.^2^ Further, a study in healthy controls only showed perfusion-related correlations with regions of haemodynamic delay rather than lead^9^. Equally, whilst studies have shown recovery of lag with reperfusion after recanalization in the hyper-acute/acute phase of stroke^10^, others investigating lag recovery in the subacute phase, have not.^6^ A second hypothesis suggests that haemodynamic lag is partly determined by the intrinsic temporal dynamics at the level of the functional neural networks^2^ and may be hard-wired by the underlying structural connectivity between brain regions.^11^ This change in lag may be in the order of 1 second, based on studies on healthy controls^2^, however extrapolating this evidence to patients with stroke can be precarious. In practice, disentangling the relative contributions of neural versus vascular processes to haemodynamic lag is challenging using fMRI alone, particularly in aging and diseased states.

A third plausible explanation for haemodynamic lag observed in non-lesioned brain, and one that will be the subject of this study is neurovascular decoupling. It is well established that stroke disrupts neurovascular coupling,^12,13^ primarily due to ischemia-sensitive glial dysfunction.^6,14,15^ The vascular response to a given change in neural activity can be affected by alterations in CVR. CVR represents the vasodilatory response of cerebral microvasculature to increased metabolic demands, quantified in response to a vasodilatory trigger such as hypercapnia.^16–20^ Specifically, CVR can be quantified as %BOLD signal change per unit change in end-tidal CO_2_ in response to hyperventilation^21^ or breath-holding^22^. The breath-holding paradigm produces robust and repeatable measures of CVR even in suboptimal performance of the breath-holding task (e.g. in patients).^16^ We have previously shown that CVR is reduced within the lesioned and perilesional tissue immediately after stroke, with no recovery into the chronic phase.^22^

In this novel longitudinal study, we directly investigate the extent to which the fMRI BOLD signal temporal lags are related to potential reductions in CVR after stroke. We compare haemodynamic lags in a cohort of patients with left hemisphere stroke, and aged-matched healthy controls, at subacute and chronic stages after stroke and during two conditions (resting-state and breath-hold). We show that despite group-level regional differences in lag across conditions, there is a significant whole-brain spatial correlation between the resting-state and breath-holding derived lag. Further, we show that the spatial correlation between lag and CVR maps is not significant in controls, nor is it significant in the lesioned hemisphere or homologous lesion and perilesional tissue contralesionally. We suggest that alterations in CVR post-stroke have negligible effects on alterations in HRF lag and argue that lag differences may be partly driven by differential neural network dynamics. Finally, we show that lag measurements are highly consistent irrespective of the reference signal chosen for lag calculation. The results of this study will improve our ability to interpret fMRI data from patients after stroke.

## Methods

The data are available from the corresponding author upon reasonable request.

### Participant recruitment

Resting-state (RS) and breath-hold (BH) fMRI data were acquired from 59 patients following a left hemisphere infarct, and 27 age and sex-matched healthy volunteers (HV). Participants performed additional language task fMRI as part of a larger study published previously.^23–25^ Inclusion/exclusion criteria are described in Supplemental Methods. Patients underwent scanning in the sub-acute (V1-Visit 1:~2 weeks), and chronic (V2-Visit 2:~4 months) phase post ictus. The National Research Ethics Service Committee approved the study. All participants provided written informed consent.

### FMRI paradigms

During the breath-hold task, participants completed six breath-hold cycles, consisting of 14s of normal breathing, 16s of paced breathing, and 15s of end-expiration breath-hold, followed by return to normal breathing.^22^ The resting-state fMRI was performed with eyes closed. All included participants confirmed they were alert post-scan.

### Neuroimaging data acquisition and pre-processing

FMRI and T1-weighted structural MRI data were acquired using Siemens Magnetom Trio3 scanner (see Supplemental Methods for details). RS and BH images were pre-processed (non-brain voxel removal, B0 unwarping, field map correction, high pass filter 0.01Hz, slice time correction, motion correction^26^) and registered to standard space using FMRI Expert Analysis Tool (FEAT) 6.0.1, from FMRIB’s Software Library (FSL). Six motion parameters and a CSF (cerebrospinal fluid) timecourse were regressed from the pre-processed data. Outlier time-points with excessive motion were identified as >1mm movement in any direction^27^ by framewise displacement (FD) and excluded. See Supplemental Methods.

### Haemodynamic lag calculation

**Figure 1:**
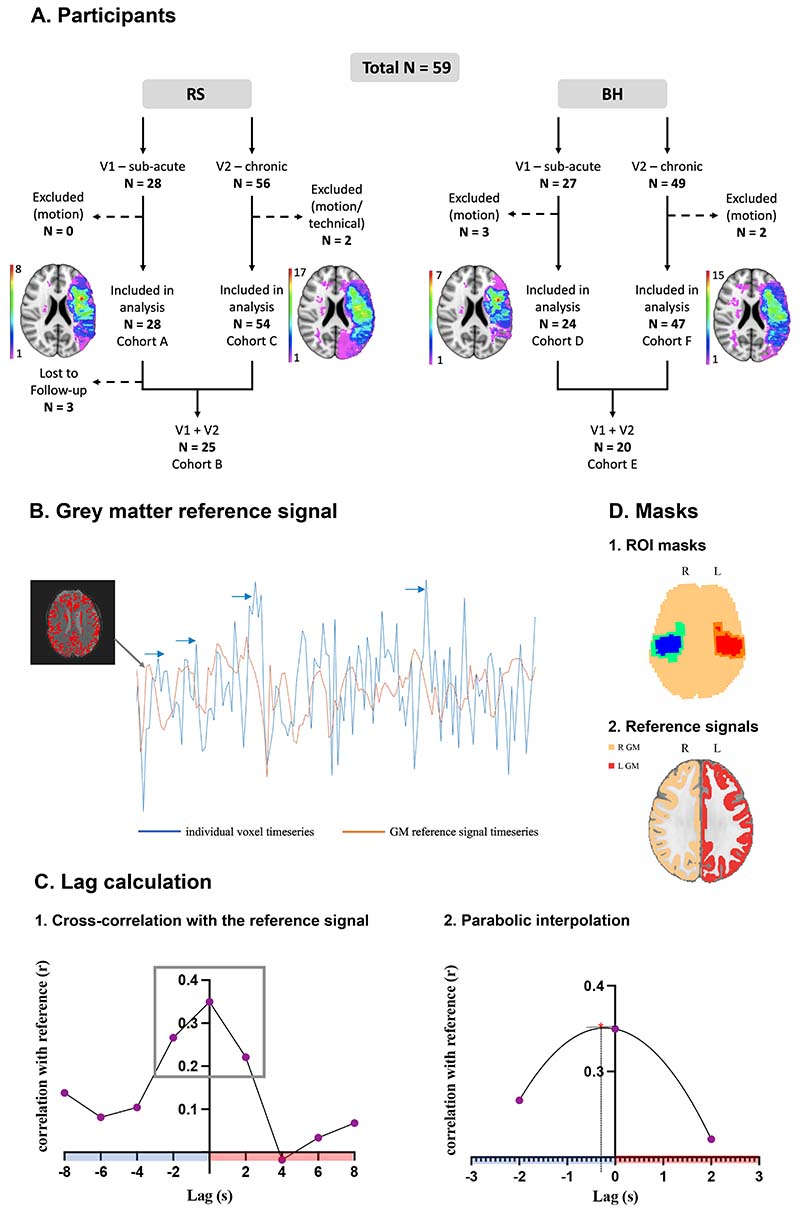
Methods **A.** Flow chart displaying number of patients included in each sub-cohort used for different fMRI lag analyses in the manuscript. All patients with BH data also had RS data, except for two exclusions due to technical issues and motion. Lesion distributions for each sub-cohort are displayed. Colour map indicates number of patients with lesion at each voxel. **B.** Lag was measured by shifting the timeseries of one voxel (blue), against that of the grey matter reference signal (orange). **C.** Correlation with reference at each shift position (left) and optimal lag calculation using parabolic interpolation (right). Positive and negative values indicate lag or lead respectively. **D.** ROI masks for one patient (1). Masks used to calculate alternative reference signals (2).

Haemodynamic lags were calculated separately for RS and BH fMRI. Six scans (1 RS and 5 BH) from patients were excluded due to excessive motion (>50% of total acquired volume). Cohort numbers included in Figure 1A represent those remaining after these exclusions. For each individual, a binary segmented grey matter mask was created from the T1 image using FAST (FMRIB's Automated Segmentation Tool)(3 classes, 4 iterations for bias field removal, 20mm FWHM smoothing), and used to derive a ‘reference’ timeseries from the pre-processed EPI images after lesion exclusion and transformation to EPI space (Figure 1B-red).

For the haemodynamic lag calculation, the Lag-suite program^6^ was adapted and used in MATLAB R2021a. Motion outliers previously saved as separate regressors were applied to pre-processed EPIs to exclude frames with excessive motion. Lag was calculated by time-shifting each voxel’s timeseries in 2-second increments by +8s (forward shift) and -8s (backward shift), representing ±4TRs, similar to previous studies.^6,7,28^ At each of the 9 shift positions, the signal was correlated with the reference signal. To prevent spurious correlations, voxels which did not display a minimum positive correlation with the grey matter signal (r<0.1) were excluded, in keeping with previous studies.^6^ The excluded voxels were unsurprisingly predominantly located within the white matter (Figure S1). For each accepted voxel, the maximum cross-correlation value of the time-shifted signal, and the cross-correlation values at the neighbouring shift positions were fitted into a parabolic function to compute the temporal shift corresponding to the maximum correlation (Figure 1C). The best-fit temporal shift, producing the maximum correlation, is the temporal haemodynamic change (lead or lag) for that voxel. Given that lag and lead are relative time latencies measured against a reference signal, rather than an absolute value, they are expressed in units of time (seconds), in keeping with previous studies.^2,6–10^

### CVR calculation

End-tidal CO_2_ traces and global signal from all participants performing the breath-hold task were inspected to ensure adequate breath-holding. In total 9 data points from 7 patients (3 at V1 and 6 at V2) were excluded from the CVR analysis due to sub-optimal breath-hold. For the remaining 21 and 41 patients at V1 and V2 timepoint respectively, CVR was calculated as %BOLD rise per unit of CO_2_.^22^ A linear interpolation was made between the end-tidal CO_2_ (sampling rate=200Hz) before and after each breath-hold and convolved with a standard double gamma variate HRF. A time-shifted CO_2_ trace, optimised for each voxel, was used as regressor in a General Linear Model (GLM) using AFNI’s 3dDeconvolve function. Voxel-wise optimisation was performed to exclude delays between the BOLD data and CO_2_ trace, caused by physiological processes or experimental setup. The CO_2_ trace was systematically time-shifted between -15s and +15s in 0.1s steps to find the best-fit delay for a given voxel, and this time-shifted trace was used as regressor in the GLM. The breath-hold response (units of %BOLD signal change per mmHg change in end-tidal CO_2_) was calculated from the beta-weight for the given voxel and regressor. (Figure.1C ‘VoxOpt’ in Geranmayeh et al^22^).

### Group-level analyses

Group-level analyses of lag maps were conducted in FSL by fitting a 2-way mixed-effect ANOVA (ordinary least squares method), to assess for a group, timepoint and interaction effect (see Supplemental Methods). False Discovery Rate (FDR) (q=0.1) was applied for multiple comparisons correction. An equivalent design was used to assess condition effect.

### Correlation of lag and CVR

A voxel-wise Spearman-rank correlation was used to assess the relationship between lag and CVR at subject-level in three tissue compartments: 1) lesion, where we have previously shown to have persistently reduced vascular reactivity after stroke^22^; 2) perilesional tissue in close proximity to the infarcted area known to display abnormal BOLD signal,^29^ hypoperfusion^30^, and reduced vascular reactivity^22^. Perilesional tissue was arbitrarily defined within 1cm of the lesion boundary; and 3) healthy tissue remote from the former two. Homologue regions of interest (ROIs) were defined within the non-lesioned hemisphere (Figure 1D–ROI mask). Voxels displaying abnormal CVR response (*r*<0.1) were excluded. Significance of correlation coefficients against 0 was assessed using a Wilcoxon signed-rank test (α=0.0083 after Bonferroni correction for 6 ROIs). To further investigate the relationship between CVR and lag, a linear mixed-effect (LME) model with fixed effects for lag was applied (only r values are reported due to inherent high between-voxel signal autocorrelation).

### Consistency of haemodynamic lag across conditions

RS and BH lag maps were spatially correlated using Pearson’s correlation, restricted to each participant’s functional space. A transformation matrix was calculated by transforming BH functional images to RS space using a rigid 3 degrees of freedom registration (FLIRT), and the resulting matrix was applied to the BH lag maps. Correlations were carried out in both groups at whole-brain level, and within ROIs including lesion, perilesional tissue, and remaining healthy tissue remote from the former two in patients. Group-level statistical analysis was conducted using Prism9.3.1 (GraphPad) and IBM SPSS Statistics24.

## Results

### Participants

Imaging data were acquired from 59 patients (PT) (36 males, mean age±SD, 61.5±12.96 years) following a left hemisphere infarct, and 27 age and sex-matched healthy volunteers (HV) (10 males, mean age±SD, 57.4±11.71 years). Due to stroke severity, only 47% of the patients were fit to undergo research fMRI in sub-acute phase (V1), therefore data were analysed in sub-groups which included different numbers of participants for each timepoint (Figure 1A and Supplemental Methods). Details of individual patients and sub-groups are provided in Tables S1 and S2 respectively.

### Group differences in haemodynamic lag

**Figure 2:**
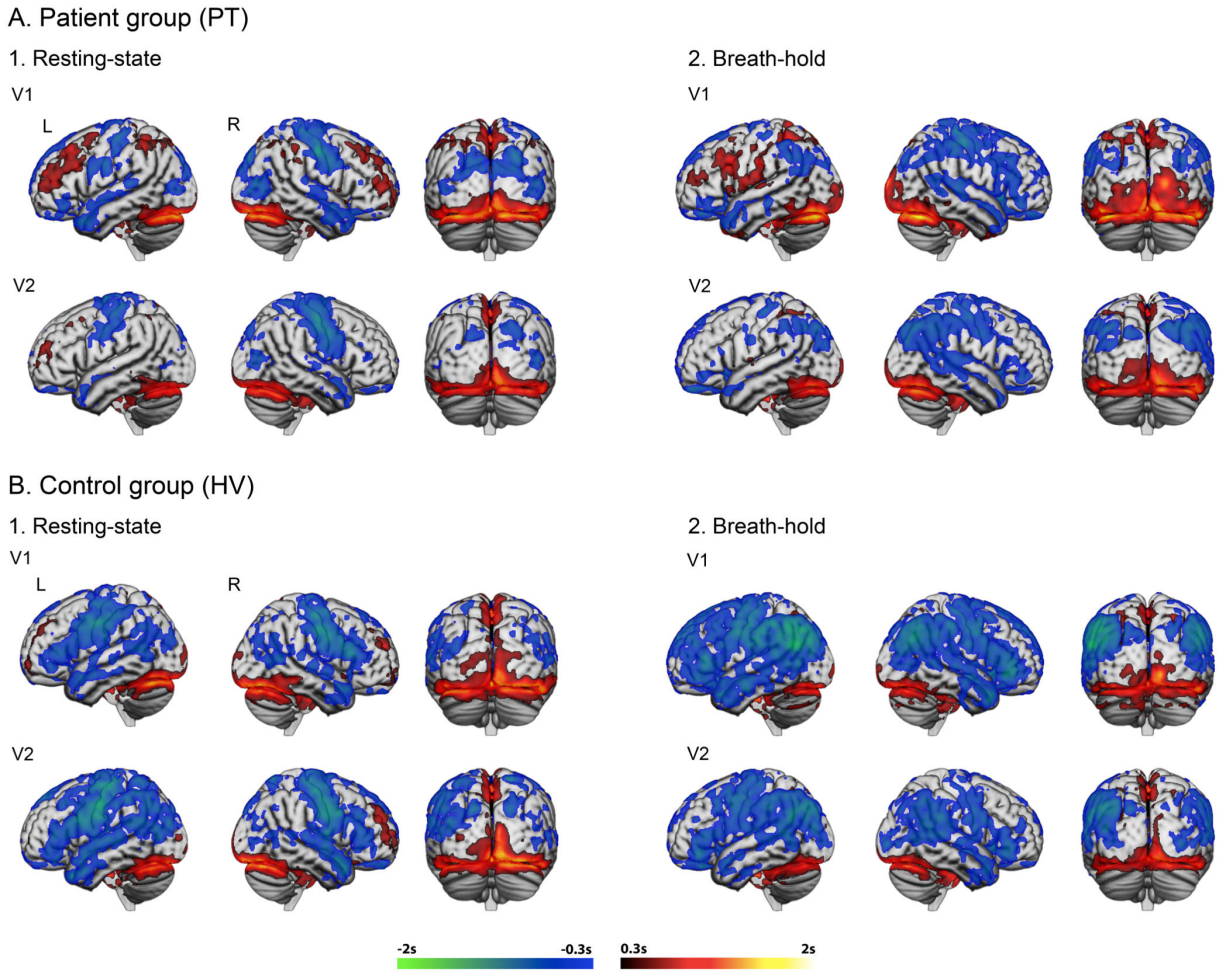
Mean grey matter haemodynamic lag (red-yellow) and lead (blue-green) greater than 0.3s in the two groups. **A.** patients; **B.** controls; Resting-state (1); breath-hold (2); sub-acute phase (V1); chronic phase (V2).

The voxel-wise group mean lag maps were calculated for both resting-state and breath-hold fMRI for each group and timepoint separately (Figure 2 and S2). Similar spatial distribution of lag was observed between groups. Resting-state data showed a relative lead (blue) in bilateral sensorimotor cortices in pre and post-central gyri, middle and superior temporal gyri, precuneous, posterior cingulate cortex (PCC), dorsal and rostral anterior cingulate and lateral occipital cortices (Table S3). The spatial extent of this relative lead appeared less pronounced in patients in the lesioned hemisphere compared to the non-lesioned hemisphere, hinting of a relative lag in patients.

In resting-state, areas of lag can be observed bilaterally in the midline occipito-parietal cortices, middle frontal gyri and posterior precuneous cortices with a significant lag attributed to the transverse venous sinuses (Table S3). A similar pattern of lag/lead was observed during BH task (Figure 2, right), with more extensive relative lead in biparietal and bifrontal regions (see also **Figure 6C**).

**Figure 3:**
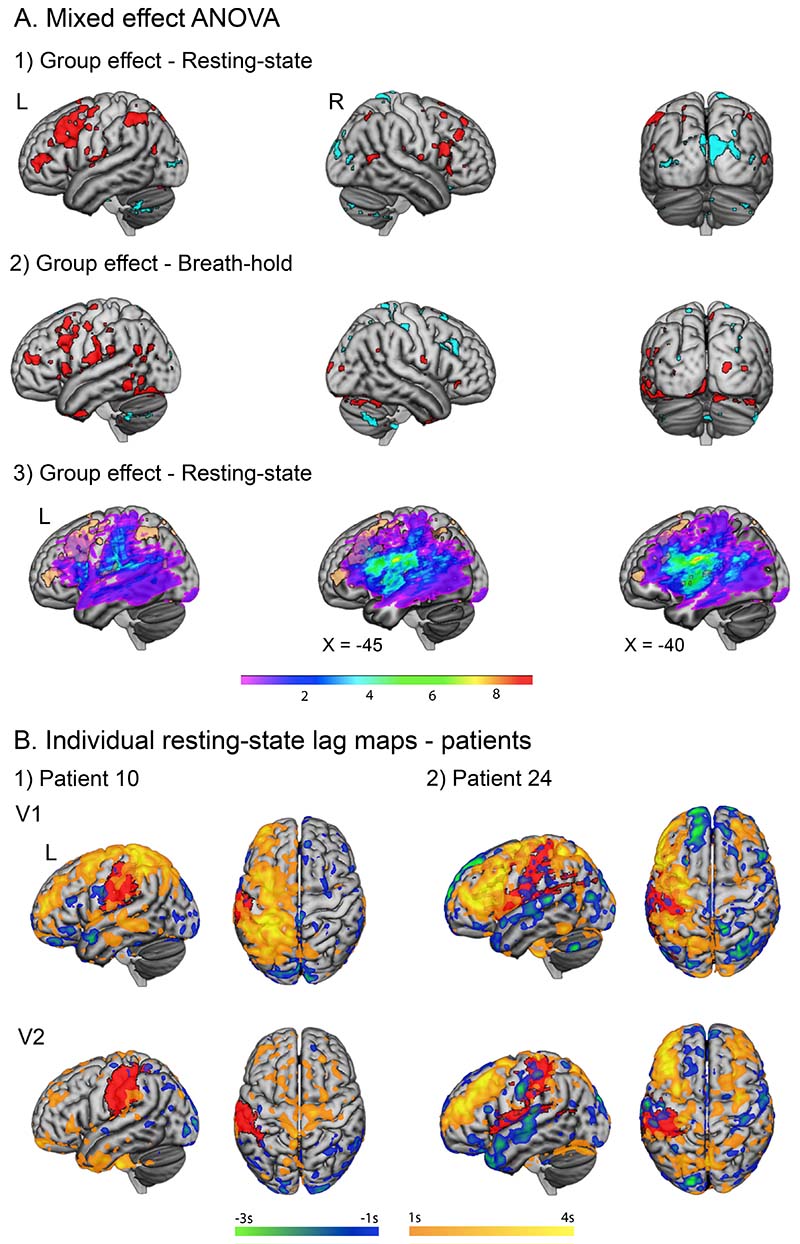
**A.** Two-way mixed-effect ANOVA results assessing for group differences in grey matter lag during RS (1) and BH (2), FDR corrected. Red and blue clusters (A.1,2) indicate areas of significantly more lag (primarily ipsilesionally) and lead respectively, in patients compared to controls. Lesion overlap (A.3) demonstrates that clusters of significant lag in patients (light brown) are found outside regions of highest lesion density. Colour map indicates group-level lesion density. **B.** RS lag maps for two example patients, sub-acutely (V1) and chronically (V2). Lag maps show lag (yellow) and lead (blue) greater than 1s. Lesions are displayed in red.

Compared to controls, patients had higher variability in lag as shown by larger voxel-wise standard deviation of the haemodynamic lag, predominantly within the lesioned hemisphere (Figure S3). Two separate mixed-effect ANOVAs were performed for each condition (N=25 RS cohort B, and N=20 BH cohort E in Figure 1A). A significant group effect on lag was observed in both conditions (Figure 3A.1-3). In resting-state, clusters of significantly higher lag in patients compared to controls were predominantly identified in the frontal and superior parietal lobes of the lesioned hemisphere, within the vascular territory of the stroke, but peripheral to areas of largest group-level lesion density (Figure 3A.3). Areas with significantly more lead in patients were observed in the bilateral occipital cortices (Figure 3A.1 coronal). Similar results were observed in the breath-holding condition, with significant lag in patients ipsilesionally in the frontal lobe, precentral and postcentral gyri, and temporal lobe (Figure 3A.2).

### Lag within the lesioned hemisphere is partially reversed over time after stroke

**Figure 4:**
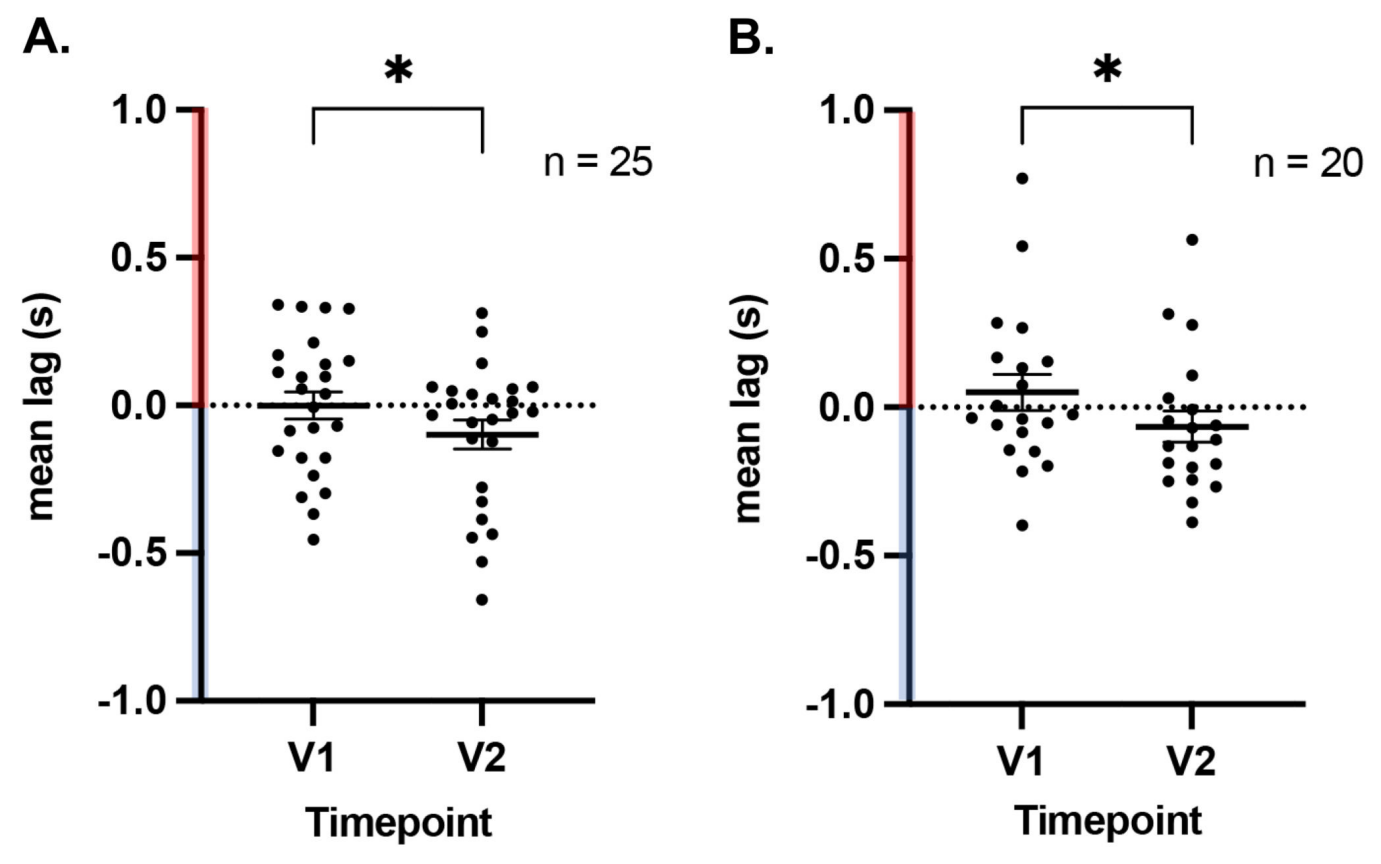
Mean resting-state (A) and breath-hold (B) haemodynamic lag in the lesioned hemisphere grey matter, in the sub-acute (V1) and chronic (V2) phase of stroke (cohorts B and E). A partial resolution of lag was observed in the chronic phase (*=*P*<0.03). Pink and blue on Y axis refer to lag and lead respectively; Mean±SEM displayed.

The extent of the lag within the lesioned hemisphere and its recovery were variable in patients (Figure S3). Figure 3B shows two representative examples with different patterns of lag recovery over time. Interestingly, patient 10 had complete occlusion of the left middle cerebral artery but showed more reduction of lag in the lesioned hemisphere when compared to patient 24, who had relatively less lag reduction over time. Despite idiosyncratic patterns of change in lag in patients, group-level calculation of mean grey matter lag within the lesioned hemisphere in cohorts B (RS-V1: -0.0001±0.23s (mean±SD); V2: -0.099±0.24s) and E (BH-V1: 0.05±0.27s; V2: -0.065±0.23s) showed a significant reduction of lag over time (one-tailed t-test *P*=0.0284 and *P*=0.0166 respectively) (Figure 4). This suggests that at group-level, across both conditions, lag in the stroke-affected hemisphere partially reduced over months after the stroke. This was driven by 14/25 and 14/20 patients in the RS and BH cohorts respectively that showed a reduction in the absolute lag value in the left hemisphere over time.

### CVR has minimal contribution to the observed fMRI lag

**Figure 5:**
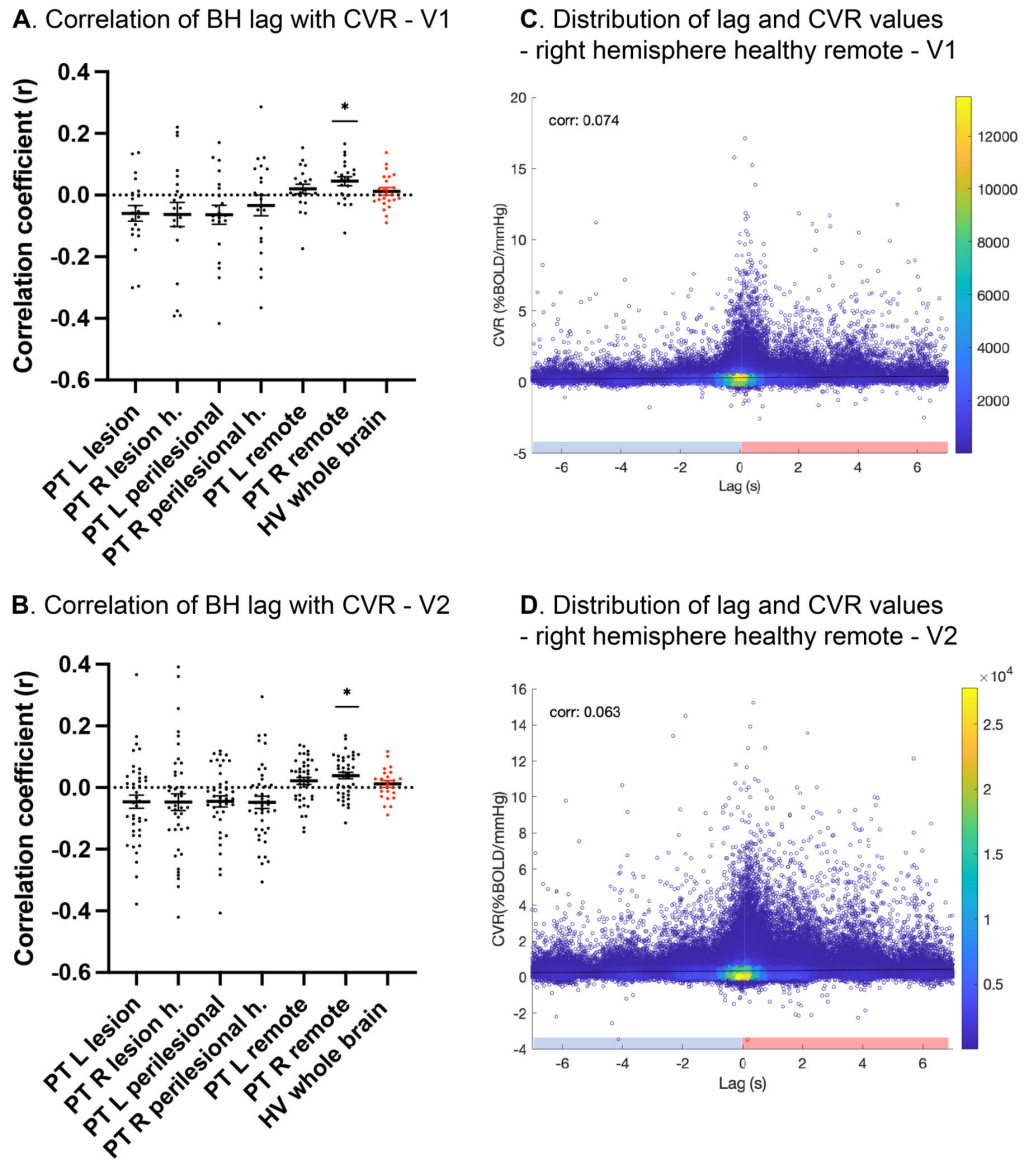
Spearman’s Rank correlation between individual participant’s lag and CVR maps, in patients (black) and controls (red), at V1 (**A**), and V2 (**B**). Mean±SEM displayed. Distribution of voxel-level lag and CVR values within healthy remote brain tissue of the right hemisphere at V1 (**C**) and V2 (**D**) shown for all patients. Colour map shows the relative density of datapoints. Pink and blue on X axis refer to lag and lead respectively.

The main purpose of this study was to investigate the relationship between fMRI lag (calculated against the grey matter reference signal) and CVR (measured separately as %BOLD signal change per unit rise in end-tidal CO_2_). Voxel-wise Spearman correlation between breath-hold fMRI lag and CVR for each participant and different ROIs are shown in Figure 5 (also see Figure S4 for absolute CVR values). There was no significant correlation (mean *r* range=-0.06 to 0.02, Wilcoxon signed-rank *P*>0.01, *α*=0.0083 Bonferroni correction) between lag and CVR within the lesion and perilesional tissue and their homologous right hemisphere regions, or healthy brain tissue remote from the lesion in the left hemisphere, at either timepoint (Figure 5A and 5B). Right hemisphere brain tissue remote from the lesion homologue showed minimal, nevertheless significant, correlation between lag and CVR at the two timepoints (V1 subject-level voxel-wise spatial correlation mean *r*<0.05, Wilcoxon signed-rank *P*<0.01; V2 mean *r*<0.05, *P*<0.001). No significant correlation was observed in controls (Wilcoxon signed-rank *P*>0.3).

A linear mixed-effect model (Figure 5C-D) confirmed very small fixed-effect coefficients in the right remote healthy tissue (V1:0.0139; V2:0.0145) suggesting that very little variance in lag is explained by CVR.

### FMRI lag is spatially correlated across brain conditions, with some regional differences

**Figure 6:**
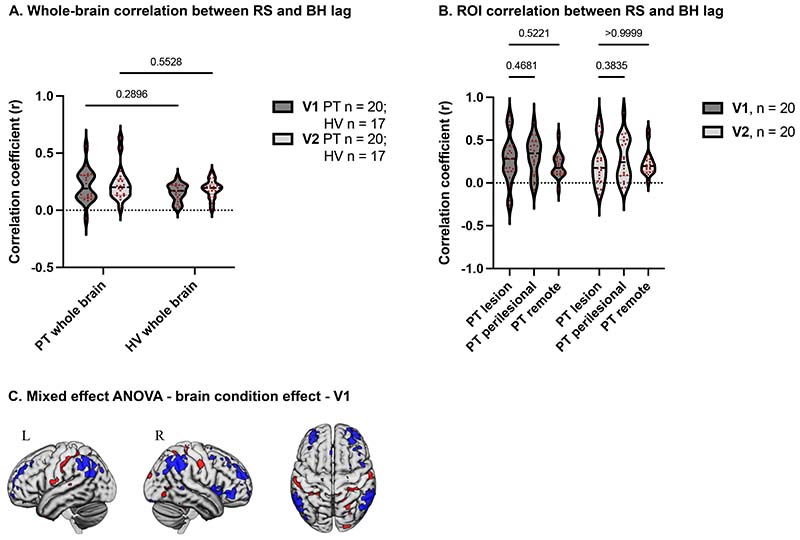
There was a consistent similarity of lag maps derived from two brain states in patients and controls **(A)**, and within ROIs in the patient group **(B). C.** Two-way mixed-effect ANOVA assessing differences in lag across brain conditions (V1), FDR corrected. Blue and red clusters show a significantly greater lead and lag respectively, in BH compared to RS.

Within participants (N=20 PT cohort E and 17 HV), lag maps derived from the two conditions were correlated across both groups and timepoints (Figure 6A). Mean subject-level voxel-wise spatial correlation were *r*=0.21 (PT-V1) and 0.23 (PT-V2) *r*=0.16 (HV-V1) and 0.19 (HV-V2), all *P*<0.0001 one-sample t-test. Similar significant correlations were observed within individual ROIs chosen in relation to lesion in patients (Figure 6B) (one sample t-tests P<0.0007). A two-way repeated measure ANOVA was further performed to assess for an effect of group and timepoint on the spatial correlation between lag maps derived from the two conditions. Results showed no significant effect of group (*F*(1,35)=1.872, *P*=0.18), timepoint (*F*(1,35)=3.158, *P*=0.0842) or interaction (*F*(1,35)=0.3053, *P*=0.5841), suggesting consistency of the similarity of lag maps in the two conditions (Figure 6A). Similarly, ROI analysis in the patient group showed no significant effect of region (*F*(1.552,29.5)=2.973, *P*=0.0782), timepoint (*F*(1,19)=0.3044, *P*=0.5876) or interaction (*F*(1.973,37.50)=1.72, *P*=0.1932) on the spatial correlations (Figure 6B).

Given all above, the lag maps across the two conditions were generally spatially similar. Nevertheless, a 2-way mixed-effects ANOVA (Figure 6C) revealed regional differences in lag between brain conditions. Clusters where the breath-holding condition showed increased lead compared to resting-state were starkly similar to regions commonly coactivated as part of the multiple demand cortex^31^ and frontoparietal attentional networks^32^, including the bilateral anterior and dorsolateral prefrontal cortex (PFC), mid-cingulate and right anterior insula/frontal operculum (aI/fO) cortices, and bilateral inferior parietal lobules (IPL) (Figure 6C, Table S4).

## Discussion

This is the first study that has directly tested the relationship between CVR and fMRI haemodynamic lag longitudinally after stroke. The results support the view that CVR has negligible contribution to the haemodynamic lag. We demonstrated no significant relationship between the two measures within the lesion and perilesional tissue, their homologous regions within the non-lesioned hemisphere, in the remote healthy tissue of the lesioned hemisphere, or indeed in controls at whole-brain level in whom CVR would be expected to be normal. Although a small but significant positive correlation was seen between CVR and lag within the remote healthy brain regions of the right hemisphere, CVR explained very little in the variability of the observed haemodynamic lag in our data, suggesting it has an overall negligible contribution to lag at a group-level in the studied cohort. To strengthen this, a longitudinal recovery of lag in the lesioned hemisphere shown in this study was not matched by a longitudinal improvement in CVR previously published in the same cohort.^22^ Thus, the observed haemodynamic latencies are likely to be related to other factors discussed below.

An alternative, but non-mutually exclusive, explanation for the lag may relate to blood flow dynamics. Although measures of haemodynamic lag and blood flow show no correlation in large cohorts of healthy controls, and regressing out CBF has been shown to have a negligible effect on these latencies^2^, patients may exhibit a different relationship. Indeed correlations between BOLD delay and several perfusion metrics have been shown after hyperacute stroke^8^ and in the subacute phase^6^. Notably however, although studies in the hyperacute stroke phase have demonstrated recovery of lag in association with recovery of perfusion, others have not been able to replicate this finding in the subacute phase of stroke.^6^ This suggests that at least in some patients, and/or at different phases of stroke, factors other than blood flow may have a greater contribution to the lag.

In this study we did not quantitatively measure CBF, but since a quarter of the patients had more than 50% vascular stenosis at the time of the study (Table S1), we were able to test for the effect of vascular stenosis on lag and infer if potentially clinically significant flow restrictions are associated with more haemodynamic lag. The lesioned hemisphere lag was similar in patients with and without 50% stenosis (Figure S5). This accords well with the observation that CBF measures only start to decrease in areas of >2s lag, (cf. 0.92s average lag in our study).^6^ Although in the absence of direct perfusion metrics we are unable to confidently exclude the contribution of blood flow restrictions to the observed lag, these results hint at the assumption that other determinants, which might include neuronal activity, might additionally contribute to the observed lag, in keeping with studies supporting a neural contribution to HRF lag.^2^

In support of the neural hypothesis, lag measured between homotopic brain regions in patients with stroke was correlated with distance from lesion as well as structural connectivity, suggesting an intrinsic neural architecture.^11^ Equally the patterns of lag observed in this cohort further support a neural hypothesis for the lag. Both patients and controls showed a similar lag distribution across brain conditions (RS, BH), despite slight regional differences. Although individual patients showed variable and idiosyncratic patterns of lag and lag recovery, the main group difference between patients and controls was increased lag in the perilesional cortices of the left hemisphere, with a lag laterality biased towards the left hemisphere. There was a partial reduction of lag in the lesioned hemisphere over time, in accord with previous studies.^6^ Without direct measurement of perfusion, it is difficult to disentangle whether group differences seen between patients and controls are due to altered perfusion, or reflect disruption of the distributed left lateralised brain networks and thus a slower information transfer within these networks. The latter was proposed as the main driving factor for lag observed in a similar cohort of patients following aphasic stroke, where the authors reported extensive behavioural correlations with lag attributed to altered function of brain networks supporting language.^7^ Although we did not find similarly extensive behavioural correlation with lag, a relative lead within the left planum temporale correlated with better fluency performance (Figure S6). A right lateralised haemodynamic signal lead in the occipital lobes in patients (Figure 3A.1), could indicate a redistribution of blood flow after the ischaemic insult in other vascular territories.^30^ Equally, it has been shown that increased occipital haemodynamic lead can be correlated with better behavioural performance in patients post-stroke, suggesting that this lead and early engagement may have a compensatory role.^7^

Symmetrical regions of haemodynamic lead were seen in the primary sensorimotor cortices and parietal lobes. Patients showed a similar pattern but a reduced spatial distribution of the haemodynamic lead in the lesioned hemisphere. This accords with previous studies showing reduced haemodynamic lead ipsilesionally^7^. Further areas of haemodynamic lead were identified in the bilateral precuneous, anterior and posterior cingulate and lateral occipital cortices, in keeping with previous studies on large cohorts of healthy controls (Mitra and colleagues^2^, Figure 2C-blue). Our findings of a relative haemodynamic lag in midline occipito-parietal cortices, middle frontal gyri and posterior precuneous cortices have also been reported^2,33^ (Mitra and colleagues^2^, Figure 2C-red).

Animal studies investigating signal propagation have identified motor, sensory and visual cortical areas as ‘sources’, where signal arises early and propagates further to posterior and anterior medial ‘sinks’.^34^ This is in keeping with our finding of a relative haemodynamic lead in somatosensory regions and could be driven by higher myelination levels facilitating earlier signal propagation.^35^ Additional imaging measures of myelination in future studies will be needed to test this hypothesis.

We showed that lag maps derived from the resting-state and breath-holding data are significantly correlated at group level, irrespective of group, time post-stroke, or region. Nevertheless, several regional differences across brain conditions were identified, most notably an increased lead in the breath-holding data (Fig 6C and Table S4) that resemble the multiple demand network^31^ and frontoparietal attentional network involved in task control.^32^ Since CVR, or indeed blood flow, is unlikely to change over the short temporal window of the fMRI session, the changes in lag latencies during breath-hold compared to resting-state must largely be neurally driven, and may reflect neural engagement of attentional networks required for execution of the breath-holding task. Similar results were found in both timepoints, with more spatially extensive clusters at the first timepoint (Table S4) perhaps reflecting the fact that performing the task for the first time is more attentionally demanding. This is in keeping with previous studies that have identified differences in lag latencies with plausible neural focality across brain conditions (eyes open vs eyes closed, before and after cued response, and diurnal states) strengthening the neural hypothesis.^2^

Patients showed highly variable lesion volumes within the left hemisphere (0.3-168cm^3^). Given our previous observation of lower CVR within the lesioned tissue^22^, we investigated the effect of lesion volume on the relationship between CVR and lag and did not find it to be a significant confound in this cohort (Figure S7). Equally, lesion volume was not a significant contributor to the number of predominantly white-matter located voxels that were excluded from lag calculations (Figure S1).

Our choice of reference signal, against which the lag is measured, is consistent with methods published previously in patients with stroke.^6,7^ We have shown that the pattern of observed lag is unlikely to be affected by the choice of reference signal. (Figure S8).

### Limitations

A limitation of this study is the inability to directly relate perfusion metrics to HRF lags. However, we did not find an association between the presence of vascular stenosis, (which can potentially cause clinically significant flow restrictions) and larger haemodynamic lags in our cohort. Future studies combining simultaneous resting-state EEG fMRI, breath-hold fMRI, and perfusion measures would be ideally placed to disentangle the complex nature of haemodynamic lag and differentiate between neural and vascular contributions to the HRF delay. Relatedly, cerebrovascular reactivity in response to hypercapnia is dependent on the arrival time of the acidic blood to the brain tissue which may be prolonged due to cerebrovascular disease, and time taken for local vasodilation of the cerebral microcirculation. Disentangling the two without time-based measures of perfusion is difficult.

A further limitation is motion which can be significant in any patient population, particularly during task. We took stringent measures to exclude data frames and subjects with excessive motion.

Although lesioned voxels were not excluded from the group-level analyses investigating between-group lag differences, it is unlikely that their exclusion would have altered the results, as the main areas of significant group differences were confined to brain regions intact in most patients (Figure 3A.3). Accordingly, the maximum area of lesion overlap (lesioned voxels in 8 patients) was in the deep white matter, away from regions of main group differences in lag.

In our study, there was a strong contribution of the transverse venous sinuses to the haemodynamic lag of highest magnitude as previously described.^2,36^

## Conclusion

Given that fMRI BOLD signal is ubiquitously used to investigate brain-behaviour relationships, disentangling the pathophysiology of the temporal delays in the BOLD signal is important for an accurate interpretation of fMRI data. This is most imperative in studies of patients with stroke where neurovascular decoupling is a frequent finding. We show for the first time that previously reported changes in CVR after stroke have a minimal contribution to any observed haemodynamic lag in the BOLD signal. We demonstrate that the temporal lag patterns are spatially correlated across conditions, with any observed differences being plausibly explained by appropriate neural focality. Further, we show that lag patterns share similarities amongst patients with stroke and controls. Future studies with concurrent EEG, fMRI and perfusion measures will be better placed to disentangle the relative contribution of perfusion versus neural processes to haemodynamic lag in patients after stroke.

## Supplementary Material

Graphical Abstract

Supplemental Publication Material
