# Supplementary figures and images for: Cerebrovascular reactivity has negligible contribution to haemodynamic lag after stroke: implications for fMRI studies
Source: Stroke. Author manuscript; Available in PMC 2023 Apr 13. (PMC7614432; doi:10.1161/STROKEAHA.122.041880)

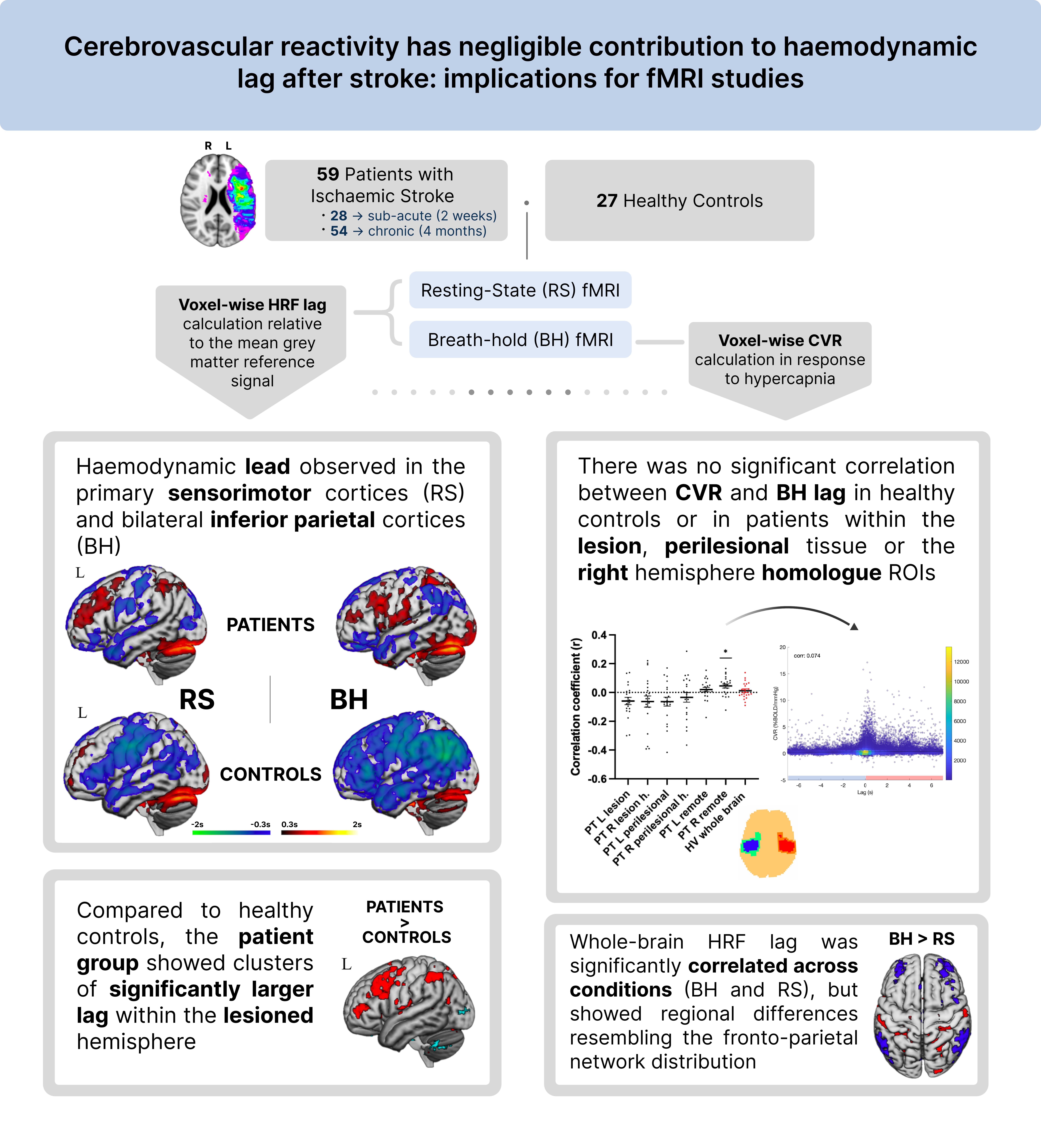

Supplement: Graphical Abstract [file EMS164669-supplement-Graphical_Abstract.jpg]
