# Supplementary material for: Cerebrovascular reactivity has negligible contribution to haemodynamic lag after stroke: implications for fMRI studies
Source: Stroke. Author manuscript; Available in PMC 2023 Apr 13. (PMC7614432; doi:10.1161/STROKEAHA.122.041880)
Supplement: Supplemental Publication Material [file EMS164669-supplement-Supplemental_Publication_Material.pdf]

## Supplemental Material

### Participants and details of cohorts studied

Resting-state (RS) and breath-hold (BH) fMRI data were acquired from 59 patients (PT) following a left hemisphere ischaemic stroke (36 males, mean age 61.5 years, range 26-80 years) and 27 healthy volunteers (HV) (10 males, mean age 57.4 years, range 37-79 years). There were no significant between-group differences with respect to age ($P$=0.1299) or sex (Fisher's exact test p=0.0615). Resting-state (RS) and breath-hold (BH) fMRI data were acquired during two visits; Patients underwent scanning in the sub-acute phase (V1-Visit 1) and chronic phase (V2-Visit 2) post-stroke corresponding to ~2 weeks and ~4 months post ictus (Mean days post-stroke for V1: 16, range 5-35; V2: 112.6, range 84-200). The mean scanning interval for the control group was 103 days, range 64-240 days). Inclusion criteria for patients consisted of left hemispheric infarct, as part of a larger study investigating recovery of language function, published previously.[23–25] Exclusion criteria included previous stroke with neurological sequelae, pre-morbid non-fluency in English language, previous neurological or psychiatric history, pregnancy, claustrophobia, inability to give informed consent, either due to conscious state, or severe receptive dysphasia. If required, patients with language impairment received additional simplified pictorial information sheet as part of the consent procedure. The breath-hold paradigm instructions were displayed in written and pictorial format. The National Research Ethics Service Committee (West London) approved the study.

Given that not all patients had technically usable fMRI data for both timepoints and scan modalities (see motion criteria below) or were unable to cooperate with the research fMRI in the sub-acute phase, and to maximise the number of participants included in each analysis, sub-group analyses included different number of participants (see Flow Diagram in Figure 1A and Supplemental Tables S1 and S2 for details and numbers of participants included in each

cohort). Resting-state data was analysed in three cohorts: cohort A included 28 patients scanned at V1 (sub-acutely), 25 of whom (cohort B) were scanned again at V2 (chronically). At V2 a total of 54 patients were scanned (cohort C), some of whom were exclusively scanned at this timepoint as they were unable to cooperate with the subacute fMRI scan at V1. Likewise, the breath-hold data was analysed in three cohorts: cohort D included 24 patients scanned at V1, of whom 20 were scanned again at V2 (cohort E). A total of 47 patients were scanned at V2 (cohort F). With the exception of 2 participants, all subjects with usable breath-hold data also had usable resting-state data (see Table S2 for cohort information).

Of the 27 controls, 23 participants had usable resting-state data again at V2. The breath-hold data was technically usable in 21 and 22 control participants at V1 and V2 respectively.

**Lesion distributions**

Lesion volume for each patient was calculated at each timepoint in standard space. For lesion distributions and volumes see Figure 1A, Supplemental Table 1 and Supplemental Table 2. Mean lesion volume was 19.86 ± 25.3 $cm^3$ (mean ± SD) at V1 (from 28 patients in RS cohort- Figure 1A –sub-cohort A), and 30 ± 38.73 $cm^3$ at V2 (from 54 patients in RS- Figure 1A – sub-cohort C). Although there was no significant difference between these two groups with respect to lesion volume ($P$=0.21), a comparison of patients scanned at V1 (N=28) with those scanned exclusively at V2 (N=29/54), revealed the former to have significantly smaller lesion volume ($P$=0.021). This is in keeping with the observation of less severe stroke (as determined by the admission NIHSS) in patients who were able to undergo fMRI imaging early after stroke in V1 compared with those who were not ($P$=0.0026).

There was a statistically significant reduction in lesion volume between the sub-acute and the chronic phase in patients with longitudinal data (cohort B; two-tailed paired t-test; $P$=0.0091, mean ± SD = 19.14 ± 25.29 $cm^3$ at V1 and 13.66 ± 16.98 $cm^3$ at V2), reflecting an expected

atrophy that is often observed in patients after stroke. Lesion volumes were highly spatially correlated between the two timepoints (*r*=0.9717).

**Neuroimaging data acquisition**

Resting-state and breath-hold fMRI data were acquired using Siemens Magnetom Trio 3 scanner, with dual-echo echoplanar imaging (EPI) sequence (TE=13 and 31 ms; TR=2 s; voxel-size=3.5x3.5x3 $mm^3$). Only the second echo was used due to better sensitivity to whole-brain BOLD. High resolution 1$mm^3$ isotropic T1-weighted structural MRI images and field maps were additionally acquired.

**Pre-processing of neuroimaging data**

Lesion masks were manually delineated by a neurologist (FG) at each timepoint on T1 images guided by clinical DWI images available acutely after stroke.

Functional images (RS and BH) were pre-processed and registered to standard space using FMRI Expert Analysis Tool (FEAT) Version 6.0.1, from FMRIB's Software Library (FSL) (www.fmrib.ox.ac.uk/fsl). To minimise registration errors, within-subject correlations of lag maps and CVR maps were performed in single-subject space. Functional images were registered to the high-resolution structural images using Boundary-Based Registration (BBR). For the creation of group lag maps, functional images were further registered to standard Montreal Neurological Institute (MNI) 1mm brain template using 12 degrees of freedom (DOF), through FMRIB's Linear Image Registration Tool (FLIRT). An inverted lesion mask was included in the registration steps to reduce potential distortions caused by registration of lesioned tissue.

FMRI pre-processing procedure included non-brain voxel removal, B0 unwarping, field map correction, high pass filter 0.01Hz, slice time correction, and motion correction using 6 motion

parameters (MCFLIRT).[26] The 6 motion parameters, and a CSF timecourse were regressed from the pre-processed data. For the latter, a single CSF voxel of interest was manually selected for each participant, in the midpoint of the anterior portion of the right lateral ventricle. This ensured better accuracy of the CSF signal as it was selected in native space. Outlier time-points with excessive motion were identified using framewise displacement (FD), defined as >1mm movement in any direction.[27] Six scans (1 RS and 5 BH) from the patients were excluded due to excessive motion defined as more than 50% of total acquired volume. The cohort numbers included in Figure 1A represent those remaining after these exclusions.

**Group-level analyses**

Two group-level analyses were conducted in FSL by fitting a 2-way mixed effects ANOVA for each brain condition in order to test for a group (PT vs HV), visit (V1 vs V2) and interaction effect. Following FDR correction for multiple comparisons, the probability thresholds obtained for the group effect contrasts were $P$=0.00180009 and $P$=0.00171564 (for patients>controls and controls>patients respectively) for the resting-state condition, and $P$=0.00272262 and $P$=0.00110617 for the equivalent contrasts in the breath-hold condition. Only participants with measurements at both timepoints were included in this analysis (N=25 PT (cohort B – Figure 1A) and 23 HV for RS; and 20 PT (cohort E – Figure 1B) and 17 HV for the BH analysis). See Supplemental Table S2 for cohort details.

An equivalent ANOVA design was used to test for significant differences in lag across brain conditions, with contrasts designed to test for a brain condition effect (RS vs BH), a group effect (PT vs HV) and an interaction effect.

**Consistency of lag maps in relation to the choice of reference signal**

In addition to the reference signal derived using the binary segmented T1 grey matter masks in individual participants, lag maps were calculated using three further reference signals; A standard MNI grey matter mask in keeping with its use in previous studies[6,7]; The grey matter mask was further divided into a left hemisphere and right hemisphere mask (Figure 1D – Reference signal); Lesioned voxels were excluded from the whole brain reference and left hemisphere reference signal calculation. Voxels in the lesion homologue were also excluded when calculating the right hemisphere-derived reference signal in order to match the volume of reference signal masks in both hemispheres. Average lag was calculated and compared within the lesion, perilesional tissue, remote healthy brain tissue within the lesioned hemisphere, their homologous regions within the right hemisphere, and bilateral sensorimotor cortices (the latter being areas with maximal haemodynamic lead – Fig 2). Results are shown below in Figure S4.

**Voxels excluded in the lag analysis:**

The average percentage of voxels excluded from the lag analyses ranged from 20 to 25% in the groups we studied (two conditions and two time points post stroke). The spatial distribution of the excluded voxels (those with <0.1 correlation with grey matter reference signal) was unsurprisingly in the white matter (Figure S1 below). There was no significant correlation between the percentage of excluded voxels and lesion volume in any of the groups we studied (all P>0.1).

## Supplemental Figures

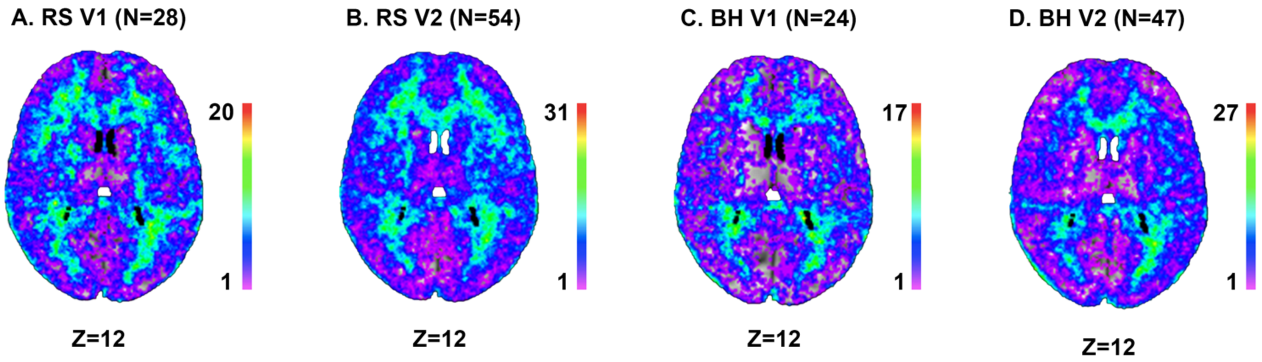


**Figure S1.**

Overlap map of voxels excluded from the lag calculation due to weak correlation (r<0.1) with the grey matter reference signal for the resting-state (A. and B.) and breath-hold (C. and D.) analyses in patients at two time points post stroke (V1: ~2 weeks ; V2: 4 months). Colour map shows the number of patients in whom each particular voxel failed to pass the 0.1 threshold. The percentage voxels excluded for each of the groups displayed was: A=25%; B= 23%; C=21% and D=20%.

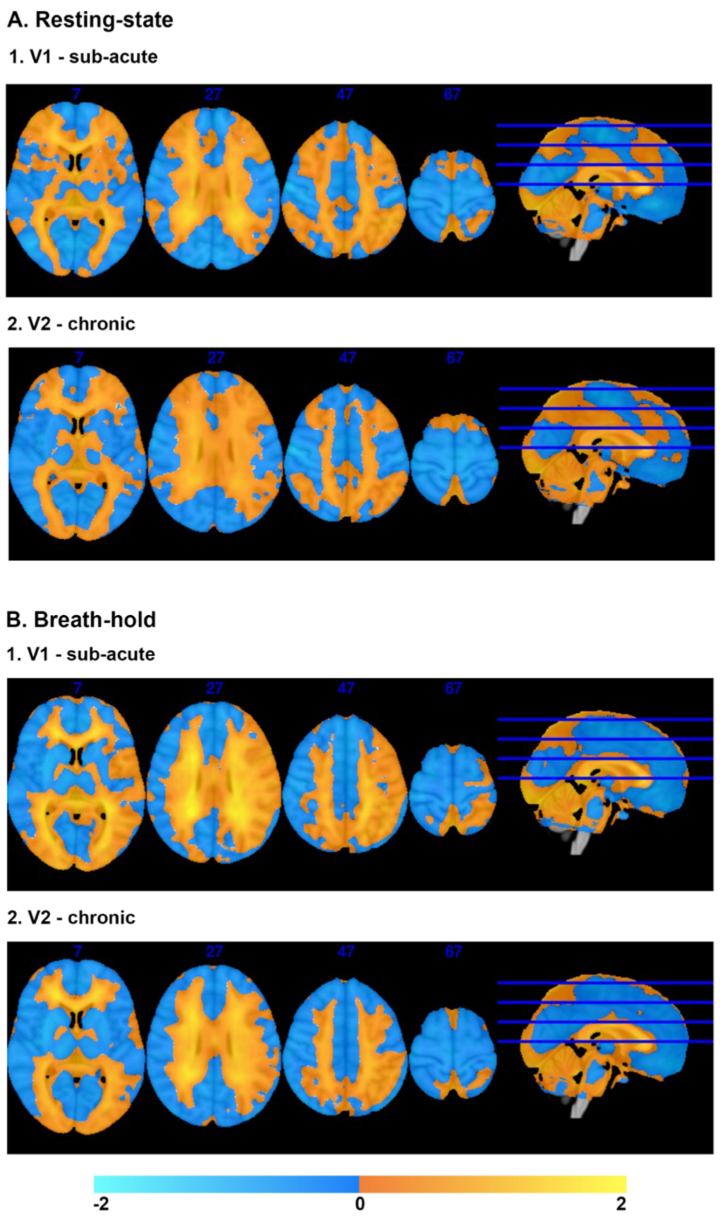


**Figure S2.**

Unthresholded volumetric representations of the mean haemodynamic lag and lead in patients for the two brain conditions (axial view) as shown in Figure 2 in the main manuscript. A. Resting-state; B. Breath-hold; sub-acute phase (1); chronic phase (2). Coordinates provided are based on volumetric results. In general, vascular structures and white matter haemodynamic latencies lagged behind those in the grey matter.

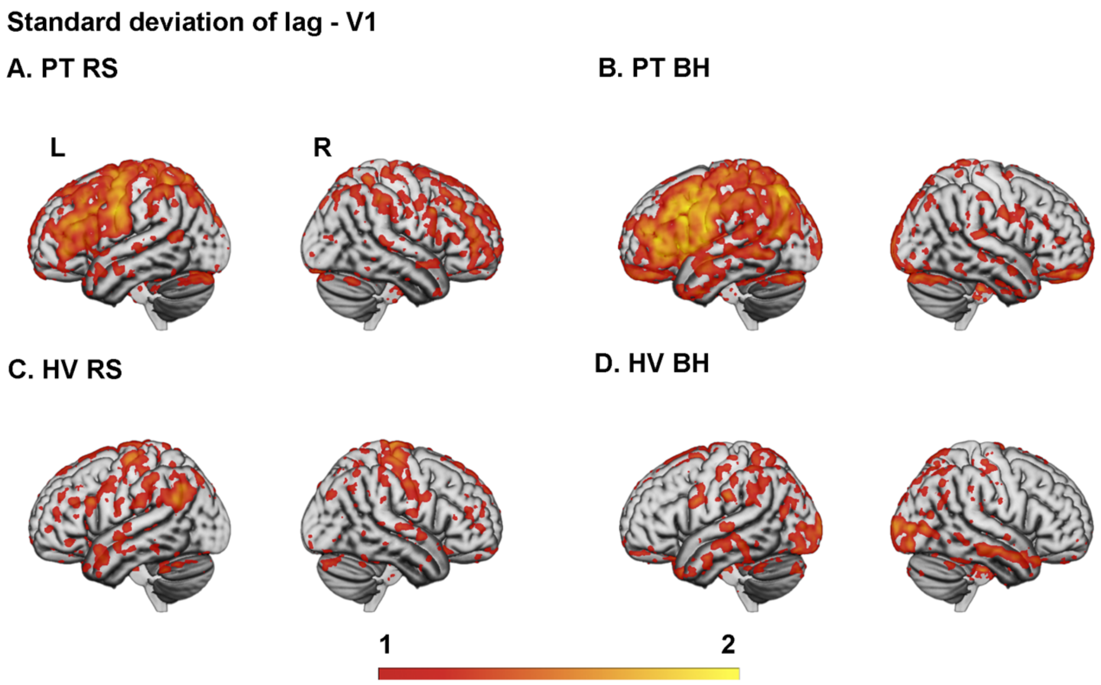


**Figure S3.**

Voxel-wise standard deviation of lag in the two conditions in patients (**A**, **B**), and healthy controls (**C**, **D**). Patients show increased variability of lag within the lesioned hemisphere compared to controls.

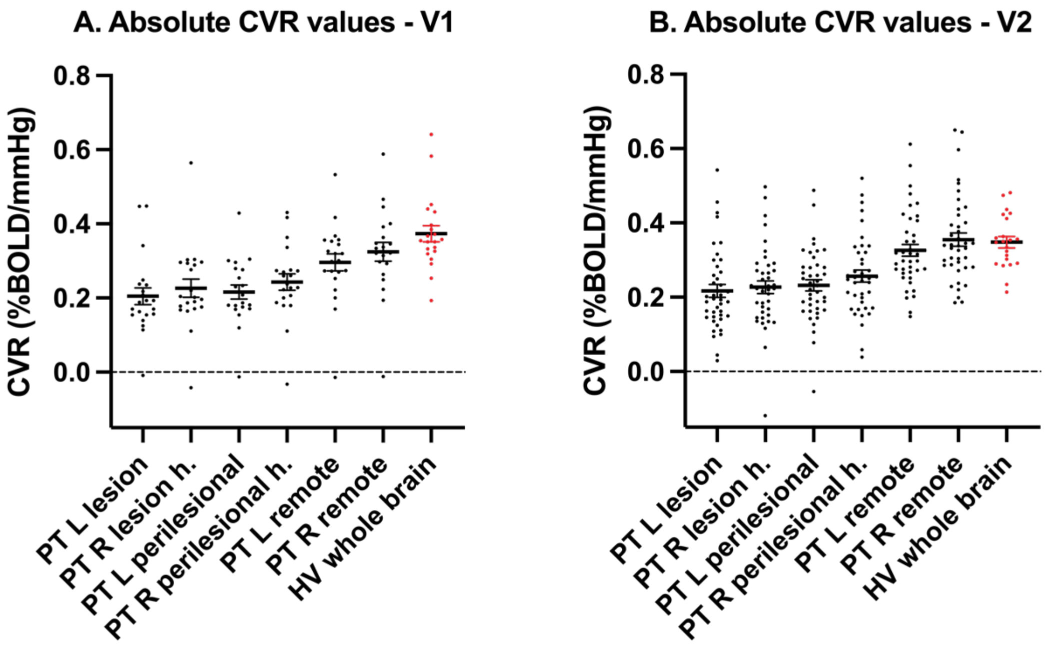


**Figure S4.**

CVR values (%BOLD rise per unit increase in end-tidal $CO_2$) averaged across each ROI mask used in the correlation of CVR with the haemodynamic lag in the patient group (PT – black), and across the whole brain in the control group (HV – red), at the first (**A.** – V1) and second (**B.** – V2) timepoints. Group mean ± SEM displayed in units of %BOLD/mmHg. There was a significant ($P$<0.001, α=0.0083 Bonferroni multiple comparisons correction) drop in CVR responses, in Perilesional tissue (V1: 0.22 ± 0.019, V2: 0.23 ± 0.015) and in Lesion tissue (V1: 0.21 ± 0.023, V2: 0.22 ± 0.017) compared to L Remote tissue (V1: 0.30 ± 0.023; V2: 0.33 ± 0.016) at both timepoints. A similar decrease in CVR between these masks was observed in the homologous ROIs in the right hemisphere at both timepoints (all $P$<0.001). Compared to the whole-brain CVR in the control group, no significant differences were observed in the CVR responses within the left or right Remote healthy tissue masks in the patient group at either timepoint ($P$>0.02, α=0.0083 Bonferroni multiple comparisons correction).

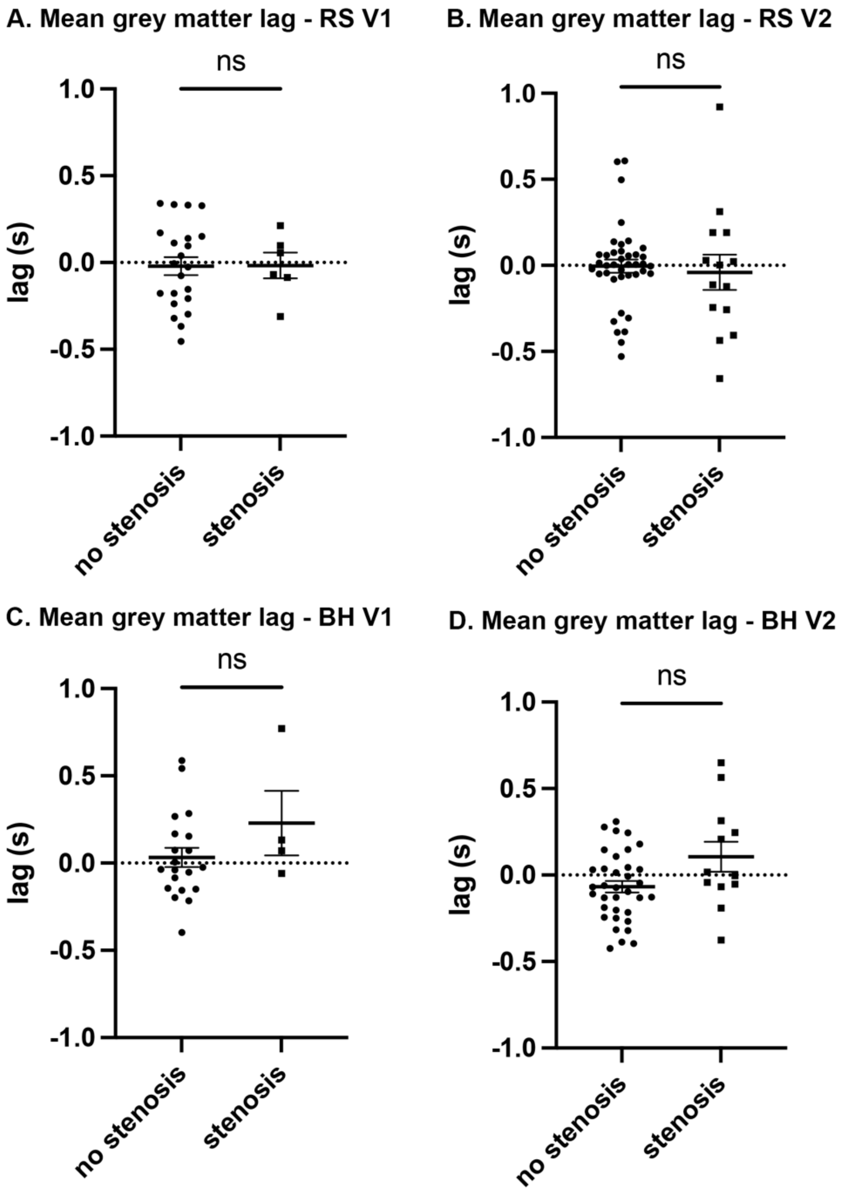


**Figure S5.**

Comparison of mean left hemisphere lag in patients with and without potentially flow limiting vascular stenosis (arbitrarily defined as >50% occlusion), in the resting-state sub-acute phase (A), and chronic phase (B), and breath-hold sub-acute (C) and chronic phase (D) after stroke. A non-parametric Mann-Whitney test showed no significant differences in the lag between the two groups (non-parametric t-test RS V1: *P*=0.9782; V2: *P*=0.5383; BH V1: *P*=0.3471; V2 *P*=0.0650). Mean ± SEM displayed.

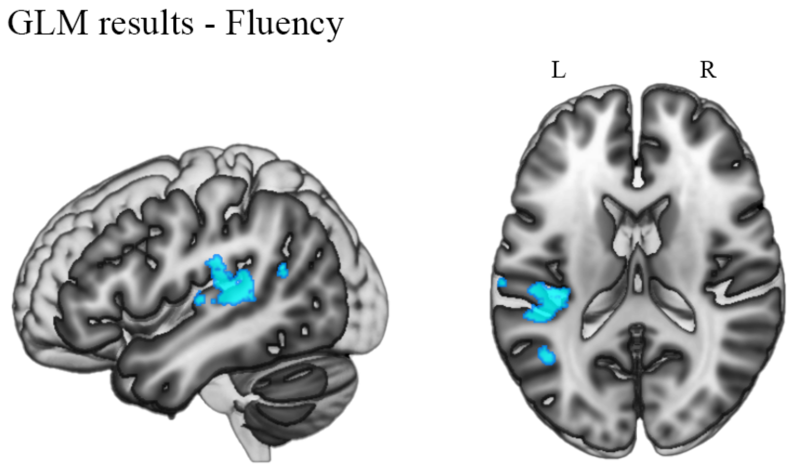


**Figure S6.**

General Linear Model assessing behavioural correlations with lag in patients at the chronic timepoint (V2). Patient showed a significant negative correlation between the BH lag and fluency scores within the left middle to posterior superior temporal gyrus (planum temporale: -46,-34,7). This suggests more lead in this region was associated with better fluency.

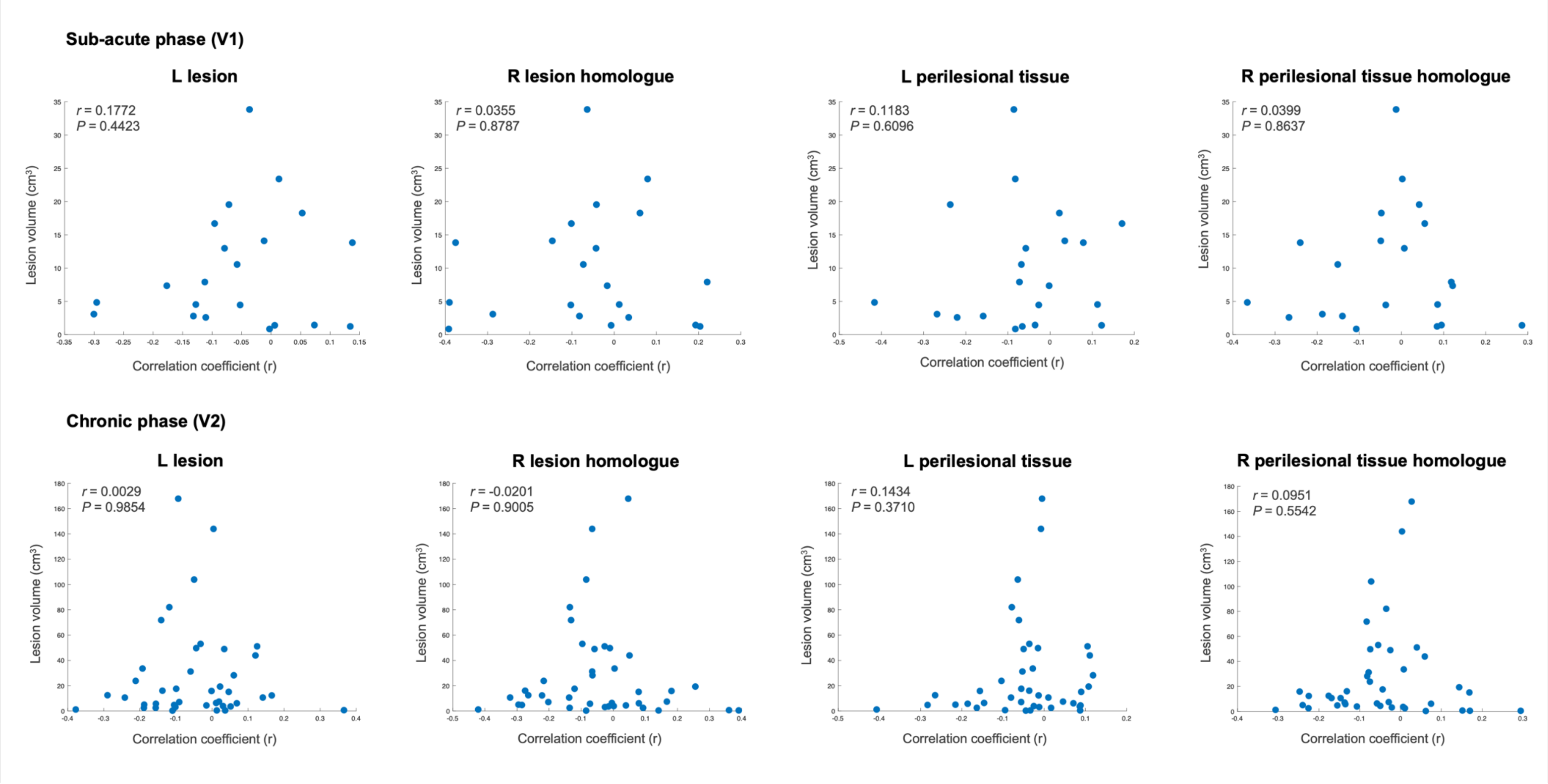


**Figure S7.**

Scatter plot showing the relationship between lesion volume and the correlation coefficients obtained for the correlation analysis of CVR with BH lag shown in Figures 5A and B. Plots are shown for separate tissue compartments (lesion, perilesional tissue, and their right hemispheric homologue

tissue) and at two time points after stroke. No significant correlation was found between lesion volume and the corresponding *r* (all $P>0.3$). Further, a whole-brain Spearman correlation analysis between CVR and BH lag at group-level, showed similar results irrespective of including the lesion as covariate (partial correlation: V1 $r=0.0499$; V2 $r=0.0446$) or not (simple correlation: V1 $r=0.0493$; V2 $r=0.0440$) (only r values are reported due to inherent high between-voxel signal autocorrelation).

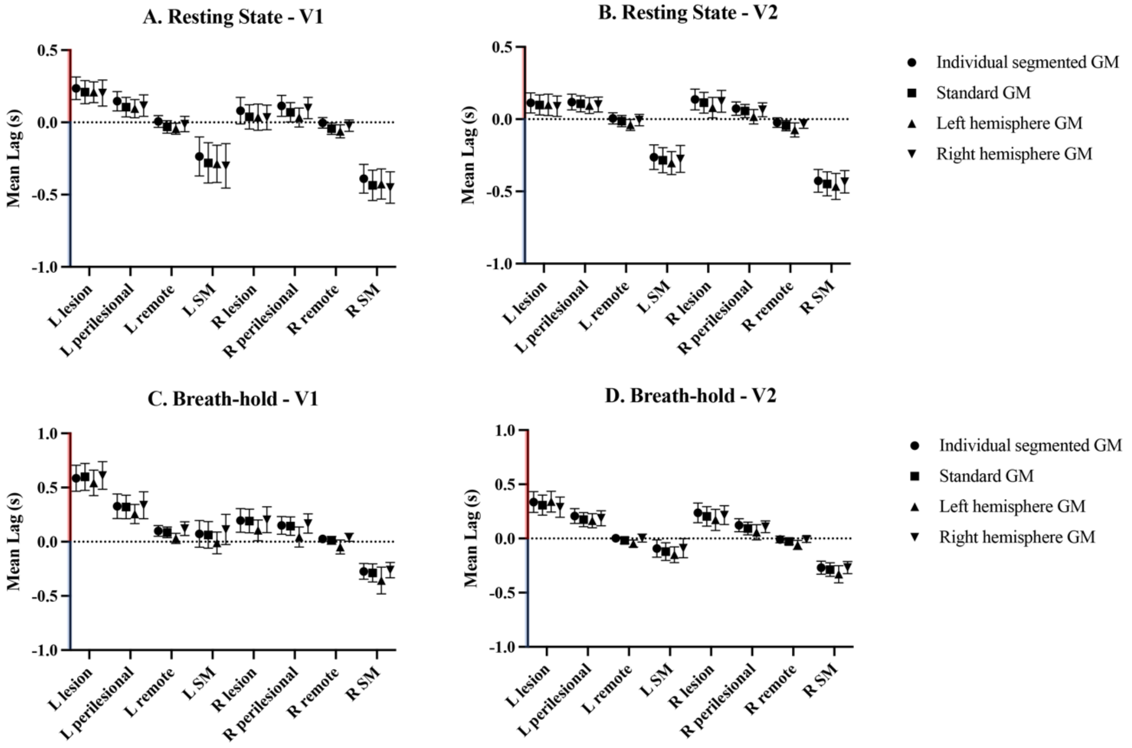


**Figure S8.**

FMRI lag is independent of the reference signal used. Mean haemodynamic lag derived from the resting-state (**A**, **B**) and breath-hold (**C**, **D**) data within 8 defined ROIs, calculated using 4 reference signals. Mean ± SEM displayed. Reference signals include that derived from the individually segmented grey matter masks (main result), standard template grey matter masks, and left and right hemisphere standard grey matter masks. Lag calculation is highly consistent across different reference signals. A two-way repeated measures ANOVA showed no significant effect of the reference signal ( RS V1: $F(1.398, 37.75) = 1.891$, $P=0.1743$; RS V2: $F(1.334, 70.72) = 1.957$, $P=0.1620$; BH V1: $F(1.129, 25.96) = 2.008$, $P=0.1676$; BH V2: $F(1.122, 51.61) = 1.573$, $P=0.2175$) and no significant interaction between the reference signal and the region of interest (RS V1: $F(4.278, 115.5) = 1.171$, $P=0.3278$; RS V2: $F(1.912, 101.4) = 0.5552$, $P=0.5680$; BH V1: $F(3.261, 75) = 0.6589$, $P=0.5922$), except in the breath-holding condition at V2 ($F(3.294, 151.5) = 4.811$, $P=0.0023$). As expected, there was a main effect of region across all groups ($P < 0.0001$).

## Supplemental Tables

| Patient | Subgroup analysis featuring the patient | Sex | Age (years) | V1 scan (days post stroke) | V2 scan (days post stroke) | Scan interval (days) | Cerebrovascular risk factors | NIHSS at admission | NIHSS at V2 | Vascular stenosis | Imaging modality used for vascular imaging | Lesion Location | Lesion Size ($cm^3$) |
|---|---|---|---|---|---|---|---|---|---|---|---|---|---|
| **1** | A, B, C, D, E, F | M | 77 | 28 | 104 | 76 | A, H | 4 | 1 | NSS | Carotid Doppler | C, SC wm (F, I) | 23.4 |
| **2** | A, B, C, D, E, F | F | 46 | 14 | 119 | 105 | eS | 3 | 0 | NSS | CTA intracranial and cervical vessels | C, SC wm (F, I) | 2.8 |
| **3** | A, B, C, D, E, F | F | 77 | 11 | 144 | 133 | S | 2 | 0 | NSS | Carotid Doppler | SC wm, SC gm (I) | 1.2 |
| **4** | A, B, C, D, E, F | M | 50 | 12 | 102 | 90 | H, Is, D, Cl | 2 | 0 | NSS | Carotid Doppler | C, SC wm (F, I O, P) | 13.0 |
| **5** | A, B, C, D, E, F | M | 44 | 12 | 161 | 149 | Cl, S | 7 | 1 | Underwent successful endarterectomy of 90% L ICA stenosis before the study. Stenosis of R vertebral artery with full distal reconstitution with collaterals | CTA intracranial and cervical vessels | SC wm, SC gm (F, I) | 7.3 |
| **6*** | A, B, C, D, E, F | M | 46 | 15 | 200 | 185 | D, H, Cl | 1 | 1 | Short L M1 segment stenosis | CTA intracranial and cervical vessels | C, SC wm, SC gm (F, P, T, O and right F) | 13.8 |
| **7** | A, B, C, F | M | 76 | 25 | 124 | 99 | A, H | 2 | 0 | NSS | Carotid Doppler | C, SC wm (P, I) | 0.3 |
| **8** | A, B, C, D, E, F | M | 60 | 10 | 127 | 117 | D, H, Ti, Cl | 2 | 1 | NSS | Carotid Doppler | SC wm (F) | 4.8 |
| **9** | A, B, C, D, E, F | M | 56 | 17 | 96 | 79 | D, H | 5 | 1 | NSS | Carotid Doppler | C , SC wm (P, F, T) | 14.1 |
| **10 *** | A, B, C, F | M | 57 | 20 | 90 | 70 | S | 6 | 1 | Complete stenosis of L MCA | MRA intracranial and cervical vessels | C, SC wm (P, F) | 34.3 |
| **11 *** | A, B, C, D, E, F | M | 75 | 16 | 101 | 85 | Cl, Is | 1 | 0 | Asymptomatic L ICA 90% stenosis | CTA intracranial and cervical vessels | C , SC wm (T, O) posterior circulation | 18.3 |
| **12** | A, B, C, D, E, F | M | 65 | 6 | 101 | 95 | - | 2 | 0 | NSS | Carotid Doppler | C, SC wm (F, I) | 9.1 |
| **13 *** | A, B, C, D | M | 64 | 6 | 89 | 83 | Cl | 13 | 2 | Full occlusion of L M3 | CTA intracranial and cervical vessels | C, SC wm (I, F, P) | 33.9 |

| | | | | | | | | | | | | | |
|---|---|---|---|---|---|---|---|---|---|---|---|---|---|
| **14** | A, B, C, D, E, F | M | 64 | 12 | 96 | 84 | A, H, Cl | 1 | 0 | NSS | Carotid Doppler | C, SC wm ( F, P) | 10.5 |
| **15** | A, B, C, D, E, F | F | 39 | 20 | 91 | 71 | Ti | 0 | 0 | NSS | CTA intracranial and cervical vessels | C, SC wm (F, I) | 7.9 |
| **16 *** | A, B, C, D, E, F | M | 65 | 11 | 104 | 93 | H, I, Cl, S | 5 | 1 | Asymptomatic L vertebral 50% stenosis | CTA intracranial and cervical vessels | C, SC gm (T) | 4.5 |
| **17** | A, B, C, D, E, F | F | 49 | 18 | 88 | 70 | - | 0 | 0 | NSS | MRA intracranial and cervical vessels | C (F) | 1.4 |
| **18** | A, B, C, D, E, F | M | 53 | 5 | 102 | 97 | - | 1 | 0 | NSS | CTA intracranial and cervical vessels | C, SC wm (F) | 0.8 |
| **19** | A, B, C, F | F | 69 | 9 | 87 | 78 | H, eS, D | 4 | 1 | NSS | Carotid Doppler | C, SC wm (T,P,I) | 75.4 |
| **20** | A, B, C, D, E, F | M | 54 | 14 | 99 | 85 | Is, H, Cl | 1 | 0 | NSS | Carotid Doppler | C, SC wm (F) | 19.6 |
| **21** | A, B, C, D, E, F | M | 63 | 7 | 90 | 83 | Cl | 1 | 0 | NSS | Carotid Doppler | SC wm | 2.6 |
| **22** | A, B, C, D, E, F | M | 50 | 20 | 101 | 81 | D, H, Cl, S | 0 | 0 | NSS | Carotid Doppler | C, SC wm (I, T) | 3.1 |
| **23** | A, B, C, D, E, F | F | 48 | 17 | 95 | 78 | H, Cl, T | 10 | 4 | NSS | CTA intracranial and cervical vessels | C, SC wm (I, F, P) | 79.3 |
| **24** | A, B, C, D, E, F | F | 62 | 10 | 94 | 84 | S | 2 | 0 | left ICA endarterectomy prior fMRI | CTA intracranial and cervical vessels | C, SC wm, SC gm (P) | 8.0 |
| **25 *** | A, B, C, F | M | 67 | 35 | 90 | 55 | eS, H, A, Is | 12 | 1 | L M1 thrombus with complete occlusion | MRA intracranial and cervical vessels | C, SC wm ( I, F, T, P) | 89.2 |
| **26** | A, D | M | 75 | 14 | - | - | Cl, Is, D | 3 | - | NSS | Carotid Doppler | SC gm | 1.4 |
| **27** | A, D | F | 53 | 8 | - | - | H, A | 3 | 1 | NSS | Carotid Doppler | C, SC wm (F) | 16.7 |
| **28** | A, D | M | 60 | 14 | - | - | H | 3 | - | NSS | CTA intracranial and cervical vessels | C, SC wm (F, I) | 59.4 |
| **29** | C, F | M | 79 | - | 93 | - | Cl | 3 | 0 | NSS | Carotid Doppler | C, SC wm (T, F, P) | 2.5 |
| **30 *** | C | F | 79 | - | 94 | - | A, Cl | 6 | 3 | L M2 thrombus with complete occlusion | CTA intracranial and cervical vessels | C, SC wm, SC gm (I, F) | 6.9 |
| **31** | C, F | M | 79 | - | 118 | - | A, Is, Cl, H | 2 | 1 | NSS | Carotid Doppler | C, SC wm (I F) | 3.0 |
| **32** | C, F | M | 67 | - | 101 | - | H, Cl | 6 | 1 | NSS | MRA intracranial and cervical vessels | C, SC gm, SC wm ( F, O, P) | 17.6 |
| **33** | C, F | M | 56 | - | 126 | - | H, Is | 9 | 2 | NSS | Carotid Doppler | SC wm (F, P, T, O and right P, F) | 12.6 |
| **34** | C, F | F | 30 | - | 100 | - | - | 16 | 3 | NSS | MRA intracranial and cervical vessels | C, SC gm, SC wm (I, F, T, P) | 49.7 |
| **35** | C, F | F | 74 | - | 105 | - | H, Cl | 1 | 0 | NSS | CTA intracranial and cervical vessels | SC wm (O, P) | 5.1 |
| **36** | C, F | F | 68 | - | 91 | - | - | 2 | 0 | NSS | MRA intracranial and cervical vessels | SC wm | 4.8 |
| **37 *** | C, F | F | 74 | - | 101 | - | Cl | 4 | 1 | Moderate short M1 stenosis 50-75% | CTA intracranial and cervical vessels | SC wm (F, O, T, P) | 10.7 |

| | | | | | | | | | | | | | |
|---|---|---|---|---|---|---|---|---|---|---|---|---|---|
| **38** | C, F | F | 61 | - | 160 | - | A, S | 11 | - | L M1 thrombus with full recanalization after thrombectomy | DSA + CTA intracranial and cervical vessels | SC wm, C (F, I, T, P) | 168.0 |
| **39** | C, F | M | 66 | - | 109 | - | eS, H | 2 | 0 | NSS | MRA intracranial and cervical vessels + Carotid Doppler | C (F) | 7.5 |
| **40 *** | C, F | M | 68 | - | 189 | - | - | 26 | 13 | L ICA dissection with occlusion | MRA intracranial and cervical vessels | SC wm, SC gm, C (I, F, P, T) | 144.0 |
| **41** | C, F | M | 75 | - | 122 | - | Is, Cl, H, D, eS | 14 | 2 | NSS | Carotid Doppler | C, SC wm, SC gm, (I, F, P) | 82.0 |
| **42** | C, F | F | 54 | - | 98 | - | S | 5 | 1 | L ICA aneurysm repair prior fMRI | CTA intracranial and cervical vessels | SC wm, SC gm (T, P , F, O) | 33.7 |
| **43** | C, F | F | 26 | - | 170 | - | Cl | 13 | 3 | L M1 thrombus with full recanalization after thrombectomy | DSA + CTA intracranial and cervical vessels | C, SC wm ( F, I, P, T) | 53.0 |
| **44 *** | C, F | M | 48 | - | 94 | - | - | 16 | 6 | L ICA dissection with occlusion | CTA intracranial and cervical vessels | SC wm ( I, F, T, P) | 71.9 |
| **45 *** | C, F | F | 62 | - | 182 | - | H, eS, Cl | 5 | 1 | L ICA dissection with thrombus | CTA intracranial and cervical vessels | SC wm (F, P) | 12.4 |
| **46 *** | C, F | F | 38 | - | 105 | - | D, S, A, H, Cl | 19 | 3 | L ICA dissection | CTA intracranial and cervical vessels | C, SC wm, SC gm ( I, F, P, O, T) | 104.0 |
| **47 *** | C, F | M | 79 | - | 104 | - | Is, Ti, H, Cl, S | 2 | 1 | Asymptomatic R ICA 70% stenosis | Carotid Doppler | C, SC wm ( I, P, T, O) | 43.9 |
| **48** | C, F | M | 51 | - | 91 | - | - | 2 | 0 | NSS | MRA intracranial and cervical vessels | SC wm (right and left F and P) | 6.2 |
| **49** | C, F | F | 82 | - | 113 | - | H, A | 4 | 0 | NSS | Carotid Doppler | SC wm, SC gm (F, I, P) | 13.6 |
| **50** | C, F | M | 55 | - | 90 | - | Es, C | 10 | 4 | NSS | CTA intracranial and cervical vessels | C, SC gm, SC wm (F, P, T, I) | 28.4 |
| **51** | C | M | 56 | - | 93 | - | A, S, H | 2 | 0 | NSS | MRA intracranial vessels and Carotid Doppler | C(P) | 7.3 |
| **52** | C | F | 68 | - | 154 | - | A, H, Cl | 2 | 0 | NSS | DSA | C (F) anterior circulation | 15.4 |
| **53** | C | F | 72 | - | 134 | - | H, S | 9 | 9 | NSS | MRA intra and extra cranial and Carotid Doppler | C, SC wm, SC gm (O, T) posterior circulation infarct | 138.9 |
| **54** | C | F | 56 | - | 99 | - | Cl, H, S | 18 | 5 | NSS | Carotid Doppler | C, SC wm, SC gm (F, I) | 47.4 |
| **55** | C | M | 80 | - | 97 | - | T, Cl, Is | 2 | 2 | NSS | Carotid Doppler | C, SC wm (F) | 32.1 |
| **56** | C | M | 72 | - | 115 | - | D, H, Cl, A | 18 | 9 | NSS | Carotid Doppler | C, SC wm (F, P, T, I, O) | 96.3 |

| | | | | | | | | | | | | | |
|---|---|---|---|---|---|---|---|---|---|---|---|---|---|
| **57 *** | C | F | 77 | - | 167 | - | A, Is, H, Cl | 2 | 1 | M3 segment of LMCA thrombus | CT and carotid Doppler | C, SC wm (F, I, P, O) | 48.4 |
| **58 *** | F | M | 63 | - | 111 | - | A, D, Cl, H | 12 | 6 | 50-70% stenosis L CCA | MRA intra and extra cranial and Carotid Doppler | C, SC wm, SC gm (F, P, T) | 31.3 |
| **59** | F | M | 75 | - | 84 | - | H, S | 2 | 0 | NSS | Carotid Doppler | SC wm | 0.5 |

**Table S1:** *List of patients.* All patients had ischaemic stroke. For each patient, the sub-group to which the patient belonged for analysis purposes (See cohorts in Figure 1A) are included. To ascertain the level of carotid artery stenosis, all patients had carotid artery imaging, with a proportion of them additionally having extracranial or intracranial arterial imaging (N=35). The degree of vessel stenosis or occlusion was quantified based on the clinically available imaging data acquired at the time of stroke (either CT, MR angiograms or Doppler ultrasounds), interpreted by vascular neuroradiologists or experienced clinicians. Any patient with an arbitrary threshold of >50% occlusion was considered to potentially have flow limiting vascular stenosis and is marked with *. NS refers to no stenosis identified on clinical vascular imaging. M1-3 refer to branches of left middle cerebral artery. NIHSS, National Institutes of Health Stroke Scale (in patients who received thrombolysis, the NIHSS at 2 hours after administration was considered as the admission NIHSS); L, left; R, right; ICA, internal carotid artery; CCA, common carotid artery; MCA, middle cerebral artery; CTA, CT angiography; MRA, MR angiography; DSA, digital subtraction angiography; H, hypertension; Cl, hypercholesterolemia; Is, ischaemic heart disease; Ti, previous small cerebrovascular disease or transient ischaemic attacks; S, smoker; eS, ex-smoker; A; atrial fibrillation. Lesion location is in the left hemisphere unless stated otherwise: C, cortical; SC wm, subcortical white matter; SC gm, subcortical grey matter; I, insular; F, frontal; P; parietal, T; temporal; O, occipital.

| | | PT | HV |
|---|---|---|---|
| **Number of subjects** | Total | 59 | 27 |
| | Males | 36 | 10 |
| | Females | 23 | 17 |
| **Mean age (years)** | | 61.5 | 57.4 |
| **Age range (years)** | | 26-80 | 37-70 |
| **Subjects scanned at V1** | RS | 28 (cohort A) | 27 |
| | BH | 24 (cohort D) | 21 |
| **Subjects scanned at V2** | RS | 54 (cohort C) | 23 |
| | BH | 47 (cohort F) | 22 |
| **Subjects scanned at V1 and V2** | RS | 25 (cohort B) | 23 |
| | BH | 20 (cohort E) | 17 |
| **Average - Scanning timepoint post-stroke (days)** | V1 | 16 | - |
| | V2 | 113 | - |
| **Range - Scanning timepoint post-stroke (days)** | V1 | 5-35 | - |
| | V2 | 84-200 | - |
| **Scanning interval HV (time between visits) (days)** | | - | 103 |
| **Range - Scanning interval HV (days)** | | - | 64-240 |
| **Lesion volume (mean ± SD) ($cm^3$)** | V1 | 19.86 ± 25.3 | - |
| | V2 | 30 ± 38.73 | - |

**Table S2.** Group-level data showing the total number of participants for each group and scan condition, age, scanning interval and lesion volume.

| Cluster index | Cluster size (voxels) | Maximum lead/lag (s) | MNI coordinates of maximum intensity voxel | | | Cluster location (Harvard-Oxford Atlas) |
|---|---|---|---|---|---|---|
| **Clusters of significant haemodynamic lead – RS, V1, Patient group** | | | | | | |
| **1** | 15953 | -1.06 | 11 | -77 | 0 | (R and L) lingual gyrus, intracalcarine cortex, precuneous cortex, cingulate gyrus (posterior division - PCC), supracalcarine cortex, cuneal cortex, occipital fusiform gyrus |
| **2** | 8462 | -1.19 | 58 | -13 | 44 | (R) postcentral and precentral gyri |
| **3** | 6305 | -1.12 | -3 | -18 | 57 | (R and L) precentral and postcentral gyri (midline), supplementary motor cortex (SMA) |
| **4** | 4836 | -0.887 | 25 | -5 | -23 | (R) amygdala, hippocampus, parahippocampal gyrus, temporal pole |
| **5** | 3598 | -1.11 | 1 | 36 | -13 | (R and L) paracingulate gyrus, frontal medial cortex, cingulate gyrus (anterior division) |
| **6** | 2186 | -0.935 | 45 | -72 | 7 | (R) lateral occipital cortex |
| **7** | 1635 | -1.02 | -32 | 10 | -36 | (L) temporal pole |
| **8** | 1570 | -0.951 | -35 | -11 | -34 | (L) temporal fusiform cortex, parahippocampal gyrus, hippocampus |
| **9** | 1219 | -0.81 | 57 | -3 | -20 | (R) superior and middle temporal gyrus |
| **10** | 1173 | -0.925 | -5 | 55 | 33 | (L) superior frontal gyrus, frontal pole |
| **11** | 1085 | -0.735 | -6 | -83 | 28 | (L) cuneal cortex, lateral occipital cortex, occipital pole |
| **12** | 905 | -0.812 | -45 | 12 | -28 | (L) temporal pole |
| **13** | 817 | -0.771 | -51 | -22 | 45 | (L) postcentral gyrus |
| **14** | 543 | -0.821 | 28 | -86 | 38 | (R) lateral occipital cortex |
| **Clusters of significant haemodynamic lag - RS, V1, Patient group** | | | | | | |
| **1** | 85478 | 1.59 | 24 | -44 | 23 | (R) caudate; (R and L) thalamus, cerebral white matter |
| **2** | 38003 | 1.53 | -4 | -84 | -19 | (R and L) occipital pole, occipital fusiform gyrus, lateral occipital cortex, lingual gyrus, cerebellum |

| | | | | | | |
|---|---|---|---|---|---|---|
| **3** | 1865 | 1.07 | 3 | -43 | 77 | (R and L) precuneous cortex (superior aspect) |
| **4** | 974 | 1.23 | 0 | -45 | -27 | Cerebellum |
| **5** | 523 | 0.802 | -34 | 29 | 46 | (L) middle frontal gyrus |
| **Clusters of significant haemodynamic lead – BH, V1, Patient group** | | | | | | |
| **1** | 18941 | -1.11 | -18 | 11 | -8 | (L) putamen, accumbens; (R and L) cingulate gyrus ( anterior division), paracingulate gyrus, frontal medial cortex |
| **2** | 15193 | -1.04 | 26 | 11 | 1 | (R) putamen, caudate, accumbens, frontal orbital cortex, insula |
| **3** | 3748 | -0.758 | -6 | 25 | 46 | (L) superior frontal gyrus, paracingulate gyrus |
| **4** | 3121 | -0.855 | -5 | -14 | 52 | (R and L) SMA; (L) precentral gyrus |
| **5** | 2682 | -0.952 | 58 | -24 | 56 | (R) postcentral gyrus |
| **6** | 777 | -0.85 | 45 | 29 | 32 | (R) middle frontal gyrus |
| **7** | 527 | -0.764 | 10 | -12 | 5 | (R) thalamus |
| **8** | 508 | -0.737 | 51 | 31 | 13 | (R) inferior frontal gyrus |
| **Clusters of significant haemodynamic lag – BH, V1, Patient group** | | | | | | |
| **1** | 202143 | 2.18 | 23 | -45 | 29 | (R and L) cerebral white matter; (R) occipital pole; (L) lateral occipital cortex |
| **2** | 1733 | 1.01 | 22 | -29 | -30 | (R) cerebellum |
| **3** | 1676 | 1.01 | -4 | -63 | 67 | (R and L) precuneous cortex (superior aspect) |
| **4** | 1517 | 1.54 | 1 | -45 | -27 | Cerebellum - surrounding 4th ventricle |
| **5** | 1321 | 0.978 | -24 | -30 | -30 | (L) cerebellum |

**Table S3.** Clusters showing haemodynamic lead > 0.6s and lag > 0.6s in the patient group at timepoint 1 (V1), for the resting-state and breath-holding conditions. Only clusters larger than 500 voxels are displayed. PCC=posterior cingulate cortex; SMA=supplementary motor area; R=right hemisphere; L=left hemisphere; WM=white matter.

| Cluster index | Cluster size (voxels) | MNI coordinates of maximum intensity voxel | | | Cluster location (Harvard-Oxford Atlas) |
|---|---|---|---|---|---|
| | | | | | **Clusters of increased lead in BH compared to RS (V1)** |
| **1** | 25428 | 15 | 14 | -11 | (R and L) putamen, caudate, accumbens, thalamus, anterior insula; (R) frontal orbital cortex, inferior frontal gyrus, frontal operculum cortex (**R aI/fO)** |
| **2** | 4252 | -33 | 49 | 30 | (L) frontal pole, middle frontal gyrus **(L dlPFC** and **L aPFC)** |
| **3** | 3527 | 63 | -45 | 36 | (R) supramarginal gyrus (posterior division), angular gyrus (**R IPL),** lateral occipital cortex (superior division) |
| **4** | 2902 | 36 | 56 | 21 | (R) frontal pole, middle frontal gyrus (**R dlPFC** and **R aPFC)** |
| **5** | 1764 | 26 | 37 | -14 | (R) frontal pole, frontal orbital cortex |
| **6** | 1543 | -61 | -50 | 39 | (L) supramarginal gyrus (posterior division), angular gyrus (**L IPL)**, lateral occipital cortex (superior division) |
| **7** | 1434 | 10 | 45 | -10 | (R) frontal medial cortex, paracingulate gyrus |
| **8** | 1396 | 2 | -26 | 31 | (R) cingulate gyrus (anterior and posterior division) |
| **9** | 1288 | -5 | 33 | 34 | (L) paracingulate gyrus, superior frontal gyrus |
| **10** | 808 | 21 | 63 | -13 | (R) frontal pole |
| **11** | 805 | -19 | -59 | -20 | cerebellum |
| **12** | 760 | -1 | -25 | 33 | (L) cingulate gyrus (anterior and posterior division) |
| **13** | 735 | 39 | 19 | 40 | (R) middle frontal gyrus |
| **14** | 526 | -6 | 45 | -3 | (L) paracingulate gyrus, cingulate gyrus (anterior division) |
| **15** | 512 | 3 | 35 | 25 | (R) paracingulate gyrus, cingulate gyrus (anterior division) |
| | | | | | |
| | | | | | **Clusters of increased lag in BH compared to RS (V1)** |
| **1** | 7816 | -55 | -24 | 39 | (L) postcentral gyrus, supramarginal gyrus (anterior division), superior parietal lobule |
| **2** | 5899 | 48 | -58 | 2 | (R) middle temporal gyrus, lateral occipital cortex |

| | | | | | |
|---|---|---|---|---|---|
| **3** | 3166 | 11 | -75 | 2 | (R) lingual gyrus, intracalcarine cortex, occipital fusiform gyrus |
| **4** | 2521 | 21 | -58 | 2 | (R) precuneous cortex, intracalcarine cortex, lingual gyrus |
| **5** | 2409 | -39 | -75 | 13 | (L) lateral occipital cortex |
| **6** | 2174 | 37 | -34 | 63 | (R) postcentral gyrus, superior parietal lobule, supramarginal gyrus |
| **7** | 1668 | -35 | 14 | 24 | (L) inferior frontal gyrus, precentral gyrus |
| **8** | 1094 | -14 | -69 | 6 | (L) intracalcarine cortex |
| **9** | 997 | 60 | -13 | 41 | (R) postcentral gyrus, supramarginal gyrus (anterior division) |
| **10** | 864 | -34 | -29 | 47 | (L) postcentral and precentral gyri |
| **11** | 709 | -25 | -68 | 36 | (L) lateral occipital cortex |
| **12** | 668 | 31 | -51 | 61 | (R) superior parietal lobule |
| **13** | 524 | 5 | -27 | 64 | (R) precentral gyrus |
| **14** | 518 | 40 | -33 | -21 | (R) inferior temporal gyrus, temporal fusiform cortex |
| **15** | 517 | -27 | 16 | -41 | (L) temporal pole |
| **16** | 508 | 37 | -11 | -25 | (R) temporal fusiform cortex, parahippocampal gyrus |
| | | | | | |
| **Clusters of increased lead in BH compared to RS (V2)** | | | | | |
| **1** | 3602 | 7 | -7 | 9 | (R) thalamus |
| **2** | 3340 | 24 | 11 | 3 | (R) putamen |
| **3** | 2130 | -15 | 18 | -8 | (L) accumbens, putamen, caudate |
| **4** | 2061 | -2 | -43 | 25 | (L) cingulate gyrus (posterior division), precuneous |
| **5** | 1605 | 2 | -41 | 28 | (R) cingulate gyrus, precuneous |
| **6** | 1501 | 62 | -45 | 38 | (R) supramarginal gyrus, angular gyrus |
| **7** | 1145 | 3 | 53 | -5 | (R) paracingulate gyrus, frontal medial cortex, frontal pole |
| **8** | 768 | 31 | 48 | 37 | (R) frontal pole |
| **9** | 584 | -2 | 54 | -9 | (L) frontal medial cortex, frontal pole, paracingulate gyrus |
| **10** | 400 | -39 | 24 | -5 | (L) frontal orbital cortex, frontal operculum cortex |
| | | | | | |
| **Clusters of increased lag in BH compared to RS (V2)** | | | | | |

| | | | | | |
|---|---|---|---|---|---|
| **1** | 15950 | -25 | -60 | 58 | (L) postcentral gyrus, superior parietal lobule, lateral occipital cortex |
| **2** | 15908 | 34 | -41 | 49 | (R) superior parietal lobule, postcentral gyrus, supramarginal gyrus |
| **3** | 12946 | 19 | -6 | -17 | (R) amygdala, hippocampus |
| **4** | 3679 | -24 | -10 | -19 | (L) amygdala, hippocampus |
| **5** | 3555 | -40 | -71 | 6 | (L) lateral occipital cortex |
| **6** | 3502 | -7 | -20 | 60 | (L) precentral gyrus, supplementary motor cortex |
| **7** | 3379 | 9 | -18 | 52 | (R) precentral gyrus, supplementary motor cortex |
| **8** | 1765 | -29 | -14 | 51 | (L) precentral gyrus, superior and middle frontal gyri |
| **9** | 1359 | 42 | -76 | 13 | (R) lateral occipital cortex |
| **10** | 877 | -42 | -47 | 13 | (L) supramarginal gyrus, middle temporal gyrus |
| **11** | 631 | 23 | -59 | 1 | (R) lingual gyrus, intracalcarine cortex, precuneous |
| **12** | 602 | -44 | 10 | -35 | (L) temporal pole |
| **13** | 580 | -59 | 9 | 31 | (L) precentral gyrus, inferior and middle frontal gyri |
| **14** | 562 | 55 | -2 | 16 | (R) central opercular cortex, precentral gyrus |
| **15** | 550 | -66 | -11 | 0 | (L) superior temporal gyrus |

**Table S4.** Clusters showing regional differences in lag between the resting-state and breath-holding condition, in the sub-acute (V1) and chronic (V2) phases. Clusters larger than 500 voxels are displayed. R aI/fO = right anterior insula/frontal operculum; dlPFC = dorsolateral prefrontal cortex; aPFC=anterior prefrontal cortex; IPL = inferior parietal lobule; R = right hemisphere; L= left hemisphere.

STROBE Statement—Checklist of items that should be included in reports of ***cross-sectional studies***

| | Item No | Recommendation | Page No. |
|---|---|---|---|
| **Title and abstract** | 1 | (*a*) Indicate the study's design with a commonly used term in the title or the abstract | |
| | | (*b*) Provide in the abstract an informative and balanced summary of what was done and what was found | p2 |
| **Introduction** | | | |
| Background/rationale | 2 | Explain the scientific background and rationale for the investigation being reported | p6 |
| Objectives | 3 | State specific objectives, including any prespecified hypotheses | p7-8 |
| **Methods** | | | |
| Study design | 4 | Present key elements of study design early in the paper | p8 |
| Setting | 5 | Describe the setting, locations, and relevant dates, including periods of recruitment, exposure, follow-up, and data collection | p8 |
| Participants | 6 | (*a*) Give the eligibility criteria, and the sources and methods of selection of participants | P8, Supplemental p1-2 |
| Variables | 7 | Clearly define all outcomes, exposures, predictors, potential confounders, and effect modifiers. Give diagnostic criteria, if applicable | p9, p10 |
| Data sources/ measurement | 8* | For each variable of interest, give sources of data and details of methods of assessment (measurement). Describe comparability of assessment methods if there is more than one group | p9, p10 |
| Bias | 9 | Describe any efforts to address potential sources of bias | p9, p10 Supplemental p3 |
| Study size | 10 | Explain how the study size was arrived at | p8, p9, p10, Figure 1A Supplemental p1, p2 |
| Quantitative variables | 11 | Explain how quantitative variables were handled in the analyses. If applicable, describe which groupings were chosen and why | p11, p12 |
| Statistical methods | 12 | (*a*) Describe all statistical methods, including those used to control for confounding | p9, p10, Supplemental p4 |
| | | (*b*) Describe any methods used to examine subgroups and interactions | p11, p12 |
| | | (*c*) Explain how missing data were addressed | p9, p10, Supplemental p4, p5 |
| | | (*d*) If applicable, describe analytical methods taking account of sampling strategy | |
| | | (*e*) Describe any sensitivity analyses | |
| **Results** | | | |
| Participants | 13* | (a) Report numbers of individuals at each stage of study—eg numbers potentially eligible, examined for eligibility, confirmed eligible, included in the study, completing follow-up, and analysed | p12, Figure 1A, Supplemental p1-2 |
| | | (b) Give reasons for non-participation at each stage | p9, p10, p12, Figure 1A, Supplemental p1-2 |
| | | (c) Consider use of a flow diagram | Figure 1A |
| Descriptive data | 14* | (a) Give characteristics of study participants (eg demographic, clinical, social) and information on exposures and potential confounders | p12, Table S1 |
| | | (b) Indicate number of participants with missing data for each variable of interest | p9, p10, p12, Figure 1A |

| | | | |
|---|---|---|---|
| | | | Table S1, Table S2, Supplemental p1-2 |
| Outcome data | 15* | Report numbers of outcome events or summary measures | p14, p15 |
| Main results | 16 | (*a*) Give unadjusted estimates and, if applicable, confounder-adjusted estimates and their precision (eg, 95% confidence interval). Make clear which confounders were adjusted for and why they were included | p14, p15 |
| | | (*b*) Report category boundaries when continuous variables were categorized | |
| | | (*c*) If relevant, consider translating estimates of relative risk into absolute risk for a meaningful time period | |
| Other analyses | 17 | Report other analyses done—eg analyses of subgroups and interactions, and sensitivity analyses | p12, p13, p14 |
| **Discussion** | | | |
| Key results | 18 | Summarise key results with reference to study objectives | p16 |
| Limitations | 19 | Discuss limitations of the study, taking into account sources of potential bias or imprecision. Discuss both direction and magnitude of any potential bias | p20 |
| Interpretation | 20 | Give a cautious overall interpretation of results considering objectives, limitations, multiplicity of analyses, results from similar studies, and other relevant evidence | p16 - 20 |
| Generalisability | 21 | Discuss the generalisability (external validity) of the study results | p17, p18 |
| **Other information** | | | |
| Funding | 22 | Give the source of funding and the role of the funders for the present study and, if applicable, for the original study on which the present article is based | p22 |

*Give information separately for exposed and unexposed groups.

**Note:** An Explanation and Elaboration article discusses each checklist item and gives methodological background and published examples of transparent reporting. The STROBE checklist is best used in conjunction with this article (freely available on the Web sites of PLoS Medicine at http://www.plosmedicine.org/, Annals of Internal Medicine at http://www.annals.org/, and Epidemiology at http://www.epidem.com/). Information on the STROBE Initiative is available at www.strobe-statement.org.
